# The African killifish: A short‐lived vertebrate model to study the biology of sarcopenia and longevity

**DOI:** 10.1111/acel.13862

**Published:** 2023-05-14

**Authors:** Avnika A. Ruparelia, Adrian Salavaty, Christopher K. Barlow, Yansong Lu, Carmen Sonntag, Lucy Hersey, Matthew J. Eramo, Johannes Krug, Hanna Reuter, Ralf B. Schittenhelm, Mirana Ramialison, Andrew Cox, Michael T. Ryan, Darren J. Creek, Christoph Englert, Peter D. Currie

**Affiliations:** ^1^ Australian Regenerative Medicine Institute, Monash University Clayton Australia; ^2^ Department of Anatomy and Physiology, School of Biomedical Sciences, Faculty of Medicine Dentistry and Health Sciences University of Melbourne Melbourne Australia; ^3^ Centre for Muscle Research, Department of Anatomy and Physiology University of Melbourne Melbourne Australia; ^4^ Systems Biology Institute Australia, Monash University Clayton Australia; ^5^ Department of Biochemistry and Molecular Biology Monash University Clayton Australia; ^6^ Monash Proteomics and Metabolomics Facility Monash Biomedicine Discovery Institute, Monash University Clayton Australia; ^7^ Department of Biochemistry and Molecular Biology Monash Biomedicine Discovery Institute, Monash University Clayton Australia; ^8^ Leibniz Institute on Aging—Fritz Lipmann Institute (FLI) Jena Germany; ^9^ Peter MacCallum Cancer Centre Melbourne Australia; ^10^ Department of Biochemistry and Pharmacology The University of Melbourne Melbourne Australia; ^11^ Drug Delivery, Disposition and Dynamics Monash Institute of Pharmaceutical Sciences, Monash University Parkville Australia; ^12^ Institute of Biochemistry and Biophysics, Friedrich‐Schiller‐University Jena Jena Germany; ^13^ EMBL Australia, Victorian Node Monash University Clayton Australia

**Keywords:** killifish, longevity, mitohormesis, sarcopenia, skeletal muscle

## Abstract

Sarcopenia, the age‐related decline in muscle function, places a considerable burden on health‐care systems. While the stereotypic hallmarks of sarcopenia are well characterized, their contribution to muscle wasting remains elusive, which is partly due to the limited availability of animal models. Here, we have performed cellular and molecular characterization of skeletal muscle from the African killifish—an extremely short‐lived vertebrate—revealing that while many characteristics deteriorate with increasing age, supporting the use of killifish as a model for sarcopenia research, some features surprisingly reverse to an “early‐life” state in the extremely old stages. This suggests that in extremely old animals, there may be mechanisms that prevent further deterioration of skeletal muscle, contributing to an extension of life span. In line with this, we report a reduction in mortality rates in extremely old killifish. To identify mechanisms for this phenomenon, we used a systems metabolomics approach, which revealed that during aging there is a striking depletion of triglycerides, mimicking a state of calorie restriction. This results in the activation of mitohormesis, increasing Sirt1 levels, which improves lipid metabolism and maintains nutrient homeostasis in extremely old animals. Pharmacological induction of Sirt1 in aged animals was sufficient to induce a late life‐like metabolic profile, supporting its role in life span extension in vertebrate populations that are naturally long‐lived. Collectively, our results demonstrate that killifish are not only a novel model to study the biological processes that govern sarcopenia, but they also provide a unique vertebrate system to dissect the regulation of longevity.

## Graphical abstract

Aging is historically characterized by an exponential increase in mortality with age, but paradoxically, in many organisms mortality rates decline late in life. In this study, we exploit the unique advantages of the African killifish ‐ an extremely short‐lived vertebrate ‐ and not only reveal its suitability for sarcopenia research, but also highlight that the metabolic hallmarks of aging are, surprisingly, reversed during the late‐life stage.

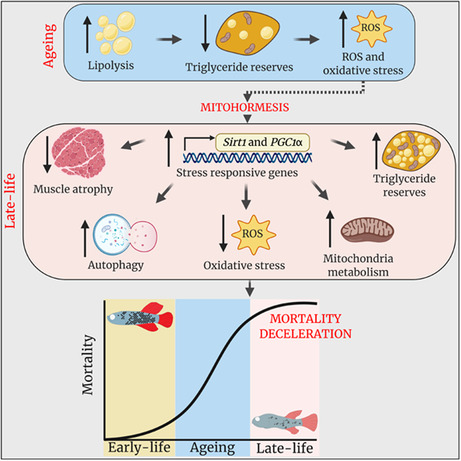

## 1 INTRODUCTION

Aging is a naturally occurring and universal phenomenon that has a devastating impact in all cells and organisms and results in a progressive decline in the ability of organs to perform their physiological functions. One such organ is skeletal muscle, which is not only indispensable for locomotion, but also serves as a critical metabolic and storage organ. Skeletal muscle aging, known as sarcopenia, is a complex and multifactorial syndrome clinically defined by the age‐related decline in muscle mass and strength. At a cellular level, sarcopenia is characterized by muscle fiber atrophy, disrupted proteolysis, stem cell dysfunction, neuromuscular junction remodeling and denervation, mitochondrial dysfunction, oxidative stress, and metabolic reprogramming among others (reviewed in Hardee & Lynch, 2019). While the stereotypic hallmarks of sarcopenia are extremely well characterized, how they contribute to muscle wasting is currently not understood. As the human population constantly challenges life expectancy barriers, a dramatic increase in the prevalence and severity of sarcopenia is expected. As such, there is a pressing need to understand the mechanisms that drive sarcopenia, following which suitable medical interventions to promote healthy muscle aging can be identified and implemented.

To study the mechanistic basis of sarcopenia, several animal models have been previously used including *Caenorhabditis elegans*, *Drosophila*, zebrafish, and rodents such as rats, mice, and guinea pigs. Most recently, a novel animal model—that of the African turquoise killifish, *Nothobranchius furzeri* (referred to as killifish from here‐in), has attracted high levels of attention in the aging fields. As a result of the ephemeral nature of their environment, killifish have evolved to exhibit the shortest known life span of any vertebrate species that can be bred in captivity. Importantly, their short life span is accompanied with canonical aging phenotypes including appearance of neoplastic lesions in the liver and gonads (Cicco et al., 2011), reduced regenerative capacity of the fin (Wendler et al., 2015), decreased mitochondrial DNA copy number and function (Hartmann et al., 2011), and shortening of telomeres (Hartmann et al., 2009) (reviewed in Hu & Brunet, 2018). While killifish display many of the hallmarks of aging, it is currently not known if they display sarcopenic pathologies, and therefore their suitability as a model for sarcopenia research remains questionable.

Therefore, in this study, we performed a thorough cellular and molecular characterization of skeletal muscle from early life, aged and extremely old late‐life stages. We reveal that many of the characteristics examined, including muscle stem cell number and muscle innervation deteriorate with increasing age, consistent with the presentation of sarcopenia. In stark contrast, we reveal a second subset of characteristics, composed of muscle fiber size and proteolysis, that deteriorated in aged fish but were found to improve in the extremely old late‐life fish. The identification of traits that reverse during the late‐life stages suggest that in extremely old animals, there may be mechanisms in place that prevent further deterioration of skeletal muscle health, which may ultimately contribute to an extension of their life span. In line with this hypothesis, we revealed, using an unbiased mathematical approach, that the late‐life stage during which we observed improved muscle health perfectly coincides with a stage with when mortality rates decline. We therefore postulate that the improvement in muscle health may be a critical factor contributing to the extension of life span in extremely old individuals.

To identify potential mechanisms for the extension in life span in extremely old late‐life animals, we examined metabolism, which has previously shown to be a strong driver of longevity. Using a systems metabolomics approach, we reveal that during aging, there is a striking depletion of triglycerides, one of the most important energy reserves in the body. Similar to the mechanisms reported for the life span extending effects of calorie restriction regimes, we observed activation of mitohormesis, a reactive oxygen species‐mediated stress resistance mechanism, in extremely old animals. We postulate that this results in improved lipid metabolism and a maintenance in nutrient homeostasis in the extremely old cohort thus providing a mechanism for their increased longevity. Collectively, our results highlight that killifish not only provide an extremely short‐lived model to study the processes that govern sarcopenia, but they also provide a novel, vertebrate system to study the biology of longevity and life span extension.

## 2 RESULTS

### 2.1 Aged killifish display muscle fiber atrophy

**FIGURE 1:**
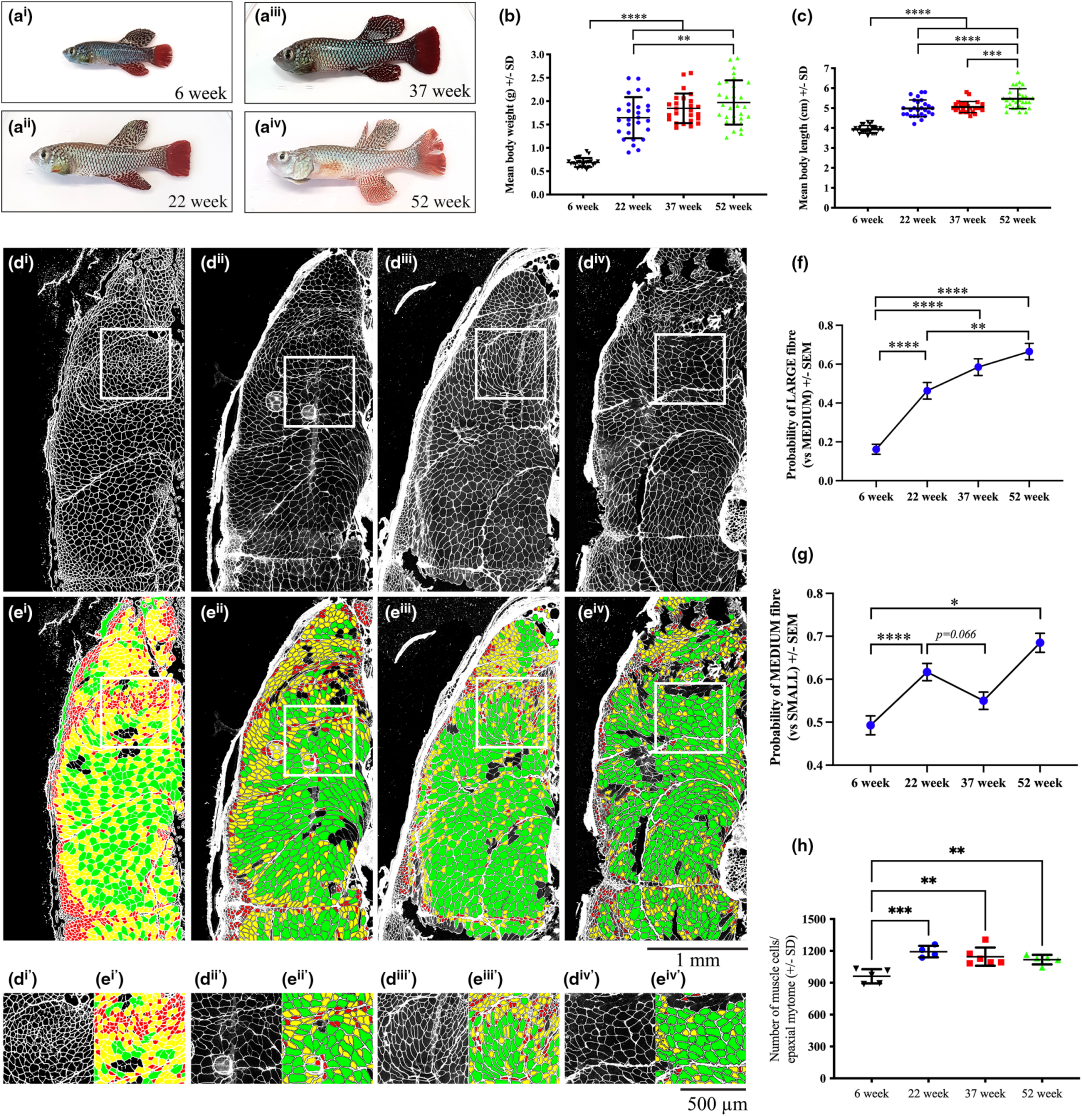
Aged killifish display skeletal muscle atrophy that plateaus in late‐life fish. (a^i^–a^iv^) Representative image of 6‐week early life, 22‐week early life, 37‐week aged, and 52‐week late‐life male killifish. Quantification of body weight (b) and body length (c) of fish from each of the four time points. Error bars represent ± SD. (d^i^–d^iv^) Representative images of Collagen labelling in 6‐week, 22‐week, 37‐week, and 52‐week old male killifish. (d^i′^–d^iv′^) Zoom in view of comparable area at each of the four time points. (e) Heat map of fiber size distribution with small fibers shown in red, medium in yellow, and large in green. (e^i′^–e^iv′^) Zoom in view of heat map of each of the four time points. (f) Probability of large (>2600 μm^2^) fibers compared to medium (between 1000 and 2600 μm^2^) fibers. Error bars represent ± SEM. (g) Probability of medium (between 1000 and 2600 μm^2^) fibers compared to small (<1000 μm^2^) fibers. Error bars represent SEM. (h) Number of muscle fibers at each of the four stages. Error bars represent ± SD. *p < 0.05; **p < 0.01; ***p < 0.001; and ****p < 0.0001 calculated using one‐way ANOVA with Tukey's multiple correction post hoc test or generalized liear mixed modelling with Bonferroni multiple correction post hoc test.

In many fish species, growth occurs throughout the life of the animal (reviewed in Ruparelia et al., 2019). To characterize the growth patterns in killifish, we examined body size at four time points across the life span of the fish: two early life stages of 6‐weeks and 22‐weeks, whereby survival is 100% and 92.3%, respectively; an aged time point at 37‐weeks, with 73.4% survival and an extremely old, late‐life 52‐week time point, whereby only 17.9% of the fish are alive (Wendler et al., 2015). From 6‐weeks to 22‐weeks of age, body weight and body length increased by 2.4‐fold and 1.2‐fold, respectively, reflective of a phase of explosive growth (Figure 1a–c). Beyond this stage, while body weight and length remained unaltered at 37‐weeks, we observed a significant increase in both parameters in 52‐week fish (Figure 1a–c). This demonstrates that unlike aged animals, early life and extremely old late‐life killifish maintain the capacity to grow continually.

To explicitly determine how muscle fiber size and number is affected with age, we stained muscle cross‐sections with an anti‐collagen antibody, which labels the extracellular matrix surrounding individual muscle fibers, and subsequently used a semi‐automated method to measure the cross‐sectional area of individual muscle fibers and determine their total counts. Muscle fibers were classified as small (red), medium (yellow), and large (green), and their spatial distribution was examined using a new computational approach based on a heat map representation (Figure 1d‐e). While small muscle fibers were predominantly found superficially, in line with these regions being the growth zones, medium and large fibers made up the deeper parts of the myotome—although a small number of small fibers were also evident in these regions. Consistent with the striking increase in body size, the probability of large muscle fibers (compared to medium muscle fibers) significantly increased with increasing age (Figure 1f). However, while the probability of medium‐sized muscle fibers (compared to small muscle fibers) significantly increased from 6‐weeks to 22‐weeks, we observed a surprising reduction from 22‐weeks to 37‐weeks, which approached significance (*p* = 0.066), but not at 52‐weeks (Figure 1g). Notably, while the number of muscle cells significantly increased from 6‐week to 22‐weeks—reflective of a phase of explosive growth—the number of muscle fibers remained unaltered after 22‐weeks, indicating that aged killifish do not undergo hypoplasia (Figure 1h). Together, this data demonstrates that aged killifish display atrophy, specifically of medium‐sized fibers, and that in extremely old late‐life fish, muscle is not only protected from atrophy but also continues to undergo hypertrophic growth.

### 2.2 Atrogin‐1 upregulation is evident in aged killifish

**FIGURE 2:**
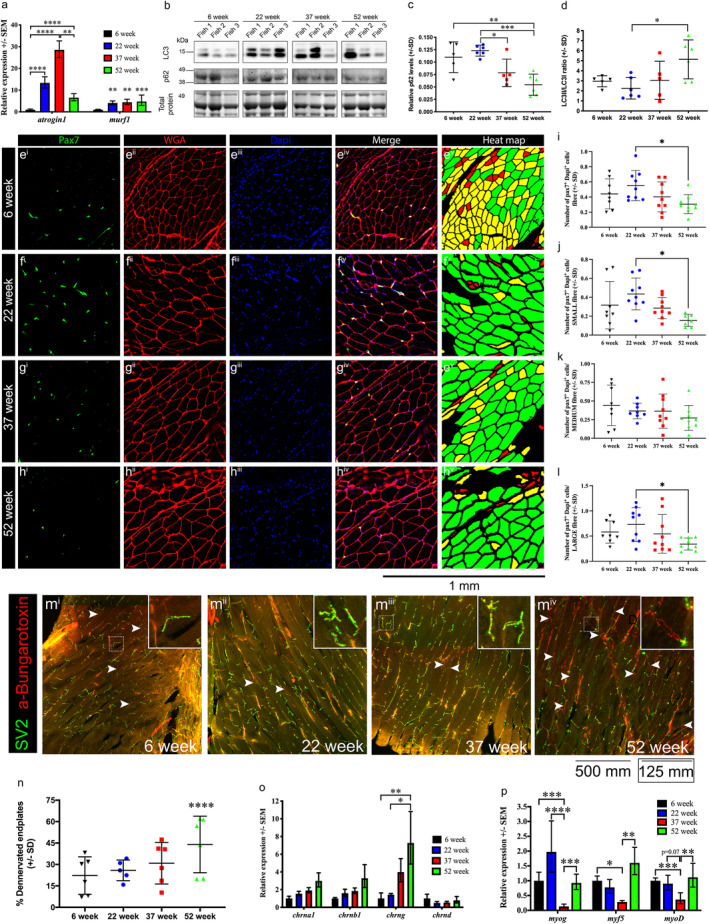
Aged and late‐life cohorts display distinct cellular and physiological characteristics. (a) qRT‐PCR analysis of *atrogin1* and *murf1* expression in 6‐week early life, 22‐week early life, 37‐week aged, and 52‐week late‐life male killifish. Levels are relative to 6‐week‐old fish and error bars represent ± SEM. (b) Western blot images for LC3 and p62, and total protein direct blue stain, on protein lysates from 6‐week, 22‐week, 37‐week, and 52‐week‐old male killifish. To accurately reflect the variation observed in LC3 and p62 levels, three represented samples from each stage are displayed. Quantification of LC3II/LC3I ratio (c) and p62 levels normalized to total protein (d). Error bars represent± SD. (e–h) Representative images of Pax7, conjugated wheat germ agglutinin (WGA) and Dapi labelling on muscle from 6‐week, 22‐week, 37‐week, and 52‐week‐old male killifish. Heat map displaying fiber size distribution is also included. (i) Total number of Pax7+/Dapi+ muscle stem cells/per fiber at each time point. Error bars represent ± SD. (j) Number of Pax7+/Dapi+ muscle stem cells/per small fiber at each time point. Error bars represent ± SD. (k) Number of Pax7+/Dapi+ muscle stem cells/per medium fiber at each time point. Error bars represent ± SD. (l) Number of Pax7+/Dapi+ muscle stem cells/per large fiber at each time point. Error bars represent ± SD. (m^i^–m^iv^) Representative maximum projection images of endplates, labelled with synaptic vesicle glycoprotein 2A (green) to detect presynaptic termini, and α‐bungarotoxin (red) to visualize acetylcholine receptors in the postsynaptic termini, in 6‐week, 22‐week, 37‐week, and 52‐week muscle. Arrowheads indicate examples of denervated endplates, which are positive for α‐bungarotoxin (red) but not synaptic vesicle glycoprotein 2A (green). Inset shows enlarged views of predominant endplates presented at each time point with 6‐week, 22‐week, and 37‐week displaying innervated endplates, and 52‐week samples displaying denervated endplates. Scale bar for inset is boxed. (*n*) Percentage of denervated endplates in 6‐week, 22‐week, 37‐week, and 52‐week‐old male killifish. Error bars represent ± SD. (o) qRT‐PCR analyses of *acetylcholine receptors*‐α (*chrna1*), −β (*chrnb1*), −γ (*chrng*), and −δ(*chrnd*) in 6‐week, 22‐week, 37‐week, and 52‐week‐old male killifish. (p) qRT‐PCR analysis of *myogenin* (*myog*), *myogenic factor 5* (*myf5*), and *myoblast differentiation protein 1* (*myoD*) in 6‐week, 22‐week, 37‐week, and 52‐week‐old male killifish. Levels are relative to 6‐week‐old fish and error bars represent ± SEM. *p < 0.05; **p < 0.01; ***p < 0.001; and ****p < 0.0001 calculated using one/two‐way ANOVA with Tukey's multiple correction post hoc test or a chi‐squared test.

A molecular hallmark of skeletal muscle atrophy is the upregulation of atrogenes: *atrogin1* (*MAFbx*, *FBXO32*) and m*urf1* (*TRIM63*) (reviewed in (Bodine & Baehr, 2014)). Atrogenes encode E3‐ubiquitin ligases, which target other proteins for degradation through the ubiquitin‐protease system, subsequently resulting in accelerated protein degradation and muscle atrophy. Given that we observed muscle atrophy in aged killifish, we hypothesized that atrogene expression will be upregulated during this phase. To test this hypothesis, we examined the expression of *atrogin1* and *murf1* in killifish muscle samples at the four time points. Our analyses reveal that *atrogin1* levels peak in the 37‐week aged cohort (Figure 2a), and this correlates with the time point at which muscle atrophy is first evident. At 52‐weeks, however, *atrogin1* levels are significantly lower than 37‐weeks, and comparable to 22‐week old fish, potentially explaining why muscle fiber size in the extremely old late‐life stage is maintained. By contrast, *murf1* expression increases at 22‐weeks with no further change in expression evident (Figure 2a).

An alternative pathway by which proteins and damaged organelles can be cleared is by autophagy. Both, excessive and defective autophagy has been shown to lead to muscle fiber atrophy. To determine how autophagy is affected in killifish muscle, we examined the levels of LC3 and p62, two markers of autophagosomes. While protein levels were highly variable, as shown in the three samples from each time point (Figure 2b), a significant reduction in p62 levels (Figure 2b,c) and increase in LC3II/LC3I ratio (Figure 2b,d) was observed in 52‐week old fish. These results indicate that although autophagy is unaffected during the aging, it is increased in extremely old late‐life killifish. Given that increased autophagy, due to calorie restriction, exercise, reduced mitochondrial respiration, and pharmacological interventions, among others, has been shown to promote longevity (Hansen et al., 2018), we hypothesized that in the extremely old late‐life fish, the higher autophagic rates may have a beneficial role.

### 2.3 Hallmark features of muscle aging are evident during late‐life but not aging

To further characterize aged and late‐life muscle biology, we examined two characteristics known to deteriorate with age in mammalian muscle. We predicted that both these characteristics would worsen in the aged cohort. The first characteristic we examined is the number of Pax7+ muscle stem cells, which are not only depleted with age but also display impaired function, subsequently hampering the regenerative capacity of muscle (Chakkalakal et al., 2012; Kimmel et al., 2020). For this purpose, we stained muscle cross‐sections obtained from trunk muscle anterior to the anal fin, with a Pax7 antibody, to detect muscle stem cells, Dapi, to identify the nuclei and a conjugated wheat germ agglutinin (WGA) to label the muscle fiber membrane. We subsequently used our heat map tool to identify the small, medium, and large fibers, following which we counted the number of Pax7+, Dapi+ muscle stem cells associated with 20–30 muscle cells from each of three size categories. Our analysis revealed that while there was no significant change in the total number of Pax7+ muscle stem cells between the 6‐week, 22‐week, and 37‐week killifish, a significant reduction in the 52‐week cohort, compared with 22‐week was observed (Figure 2e,f,g,h,i; Extended Data Figure 1 in Data S1). We next examined if this reduction was associated with a particular the size of muscle fiber. Our results indicate that both small and large fibers display a reduced number of stem cells, while the number of stem cells associated with medium fibers remains unaffected with age (Figure 2j,k,l).

An additional, well‐characterized, change that occurs during skeletal muscle aging is the deterioration of neuromuscular junctions, and denervation of aged muscle (Liu et al., 2017; Valdez et al., 2010), which subsequently compromises neurotransmission, impairs muscle contraction, and results in age‐related muscle weakness. To determine if denervation occurs with age in killifish muscle, we obtained longitudinal sections from trunk muscle anterior to the anal fin, and stained muscle endplates with a synaptic vesicle glycoprotein 2A (SV2) antibody, to identify presynaptic vesicles, and with α‐bungarotoxin to label postsynaptic termini. We subsequently quantified denervated endplates identified by the loss of presynaptic SV2 staining, but the presence of postsynaptic α‐bungarotoxin staining. In contrast to our hypothesis, muscle denervation was unaffected in the 37‐week aged cohort but instead, we found a significant increase in the percentage of denervated endplates at the 52‐week extremely old late‐life stage (Figure 2m,n). To molecularly validate this finding, we examined the expression of nicotinic acetylcholine receptor genes and myogenesis‐related genes, which characteristically increase with denervation (Wu et al., 2014). Indeed, 52‐week late‐life fish were found to display increased expression of nicotinic acetylcholine receptor γ (*chrng*), although the expression of subtypes α (*chrna1*), β (*chrnb1*), and δ (*chrnd*) remained unchanged (Figure 2o). A 52‐week late‐life fish also displayed increased expression of *myogenin*, *myogenic factor 5* (*myf5*), and *myoblast differentiation protein 1* (*myoD*) compared to 37‐week aged fish (Figure 2p), which collectively confirm that muscle denervation occurs in the extremely old fish but not aging phases. In line with the striking denervation observed in this study, previous analyses using the shorter lived GRZ strain have revealed that aged killifish display a significant reduction in locomotion (Valenzano et al., 2006). Although the locomotion capabilities of the extremely old animals in our study have not been examined, we can confirm that the animals are highly sedentary compared to younger animals. Notably, the increase in myogenic genes in the late‐life stages may also be indicative of increased fusion of muscle stem cells with existing muscle cells, which may explain the observed reduction in muscle stem cell number, and the subsequent maintenance of muscle fiber size. Intriguingly, muscle stem cells have been shown to contribute to the regeneration of neuromuscular junctions following denervation (Liu et al., 2017), and the decline in stem cell number in extremely old animals, may be also responsible for the degeneration of neuromuscular junctions during this stage.

Taken together, these findings highlight three key points: firstly, aged and extremely old killifish display many of the hallmarks of sarcopenia, supporting their use as a model for sarcopenia research. Secondly, aged and extremely old killifish display distinct phenotypes, suggesting that these two phases are physiologically different. Finally, in the extremely old animals, a combination of changes were observed, in that some characteristics deteriorate (stem cell number and muscle innervation), and others improve (fiber size and proteolysis). These results surprisingly reveal that not all aspects of muscle health deteriorate in extremely old animals and that specific pathways maybe triggered to protect the muscle from further deterioration.

### 2.4 The extremely old time point coincides with a stage at which mortality rates decrease

**FIGURE 3:**
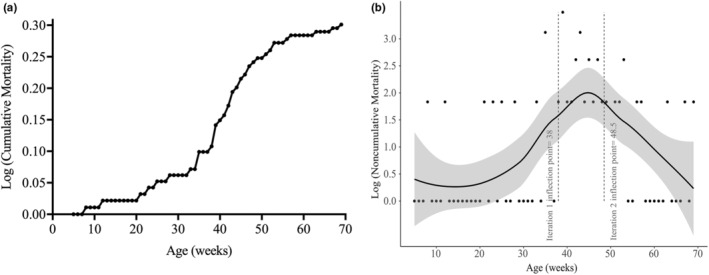
The extremely old time point coincides with a stage in which mortality rates decrease. (a) Cumulative mortality of male MZM‐0703 killifish presented on a logarithmic scale (*n* = 39). (b) Scatterplot of inflection points of the log‐scaled mortality data, as determined using the extremum surface estimator method. The smooth line is based on local regression analyses, and the grey zone around the smooth curve shows the standard error bounds, computed using a *t*‐based approximation. The two inflection points identified at 38‐ and 48.5‐weeks are shown by the dashed line.

The identification of characteristics that improve in the extremely old killifish suggest that there may be mechanisms in place that prevent further deterioration of skeletal muscle health, which we predict may contribute to an extension of their life span. To determine if the extremely old stage is accompanied with a reduction in mortality, at a population level, we reexamined survival data from our previously published life span analyses (Wendler et al., 2015). We calculated the cumulative mortality at across the life span of the fish and plotted the data on a base 10 logarithmic scale. Using this strategy, we revealed that while initially mortality rates are low, an exponential increase in mortality is evident at later stages. Importantly, this is followed by a plateau in cumulative mortality rates, supporting our hypothesis that mortality rates decrease at the extremely old stages. (Figure 3a). To mathematically identify the exact age at which mortality rates decelerate, we used the extremum surface estimator (ESE) method to look for inflection points, characterized by changes in data trajectories over time, in the mortality dataset. Briefly, the ESE works through maximization/minimization of the function that maps onto the mortality data across time, which, appropriately and in an unbiased fashion, identifies inflection points (Christopoulos, 2012, 2016). Our analyses identified two inflection points—one at 38‐weeks and a second at 48.5‐weeks, demonstrating that killifish have three distinct life‐history stages: the period prior to 38‐weeks reflecting an “early‐life” phase, 38‐weeks to 48.5‐weeks represents the “ageing” cohort whereby mortality rates display an exponential Gompertzian trend, and fish beyond 48.5‐weeks are in the “late‐life” stage of their life span whereby mortality rates plateau (Figure 3b). Importantly, mortality rates plateau at the same stage in which we observed an improvement in muscle health, thus supporting our hypothesis that the characteristics we have identified, may be critical in regulating the extension of life span in extremely old individuals.

### 2.5 Extremely old animals display a metabolic profile that is more similar to early life cohorts

**FIGURE 4:**
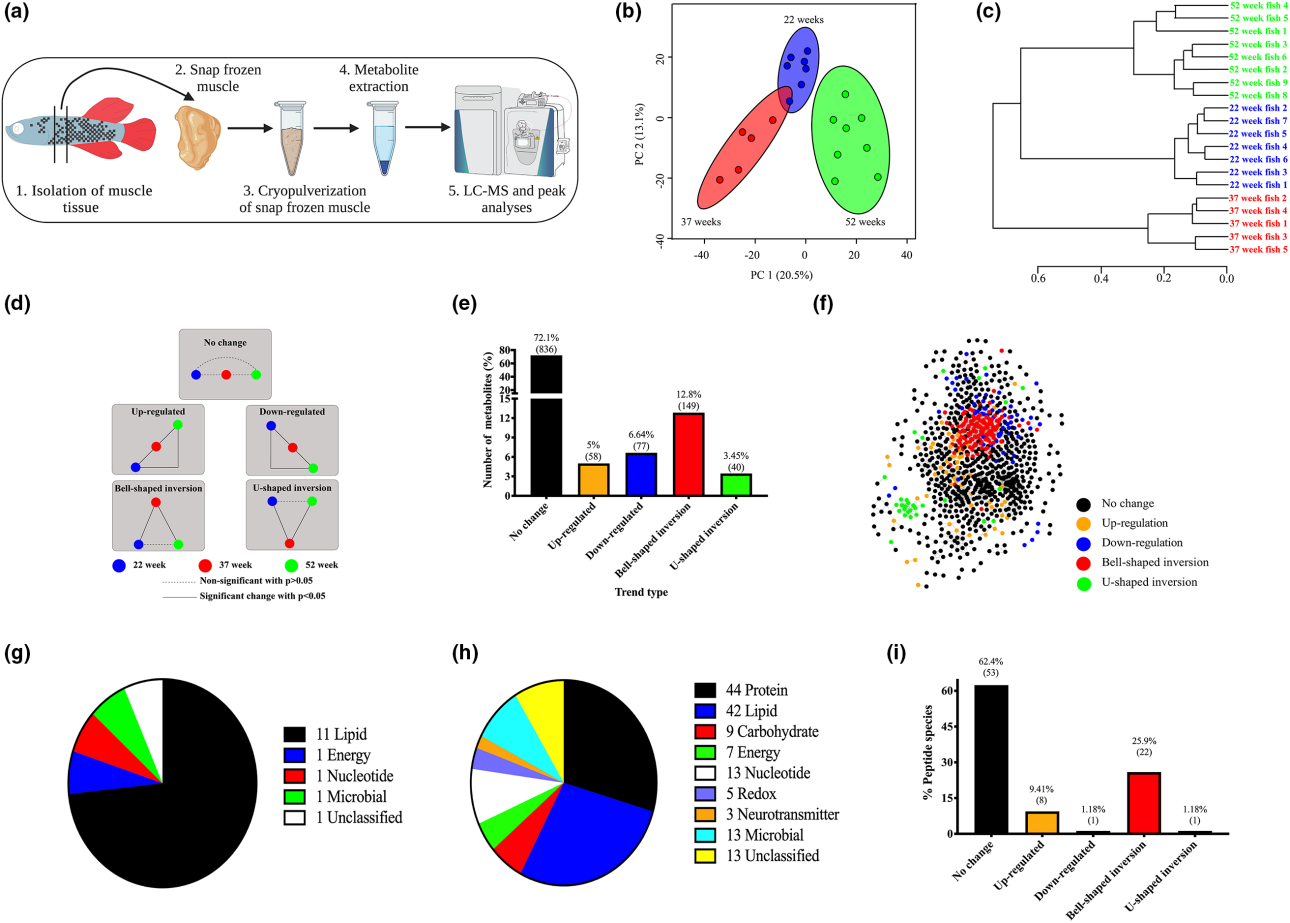
Late‐life fish display a metabolic profile that is more similar to early life cohorts. (a) Schematic of experimental design: Muscle from comparable anterior–posterior positions were extracted and snap‐frozen. Once ready for extraction, snap‐frozen muscle was cryopulverized, metabolites were extracted following which liquid chromatography coupled to high‐resolution mass spectrometry (LC–MS) was performed. (b) Principal component analyses score plot of all metabolites in skeletal muscle from 22‐week early life (blue), 37‐week aged (red), and 52‐week late‐life (green) male killifish. Clustering of samples according to age is evident. (c) Dendrogram of unsupervised hierarchical clustering analyses using spearman's rank correlation reveals 22‐week early life and 52‐week late‐life datasets are more closely related to each other than the 37‐week aged fish. (d) Schematic of five broad metabolite trend types, with 22‐week samples displayed in blue, 37‐week cohort in red, and 52‐week fish shown in green. Dashed lines represent nonsignificant changes with *p* > 0.05, and solid lines reflect significant alterations with *p* < 0.05. (e) Graph of the prevalence of each of the five trend types. (f) Network of all metabolites identified in our dataset, as determined using co‐abundance analyses. Metabolites displaying a bell‐shape inversion trend (red) are enriched in the center of the network. (g) Pie chart depicting metabolic pathways to which the top 15 most influential metabolites within the network belong to, with lipid metabolism being the most represented. (h) Pie chart depicting the enrichment of various metabolic pathways within the bell‐shaped inversion group, with protein and lipid metabolism accounting for more than half of all metabolites within this group. (i) Graph of the prevalence of peptide species across the five groups.

Given that metabolism is a strong driver of longevity and that skeletal muscle is a highly active metabolic organ, we hypothesized that metabolic alterations may provide an explanation for the differences in cell biology and longevity observed between the aged and extremely old animals. To this end, we utilized an untargeted metabolomics approach, examining muscle samples from the 22‐week early life, 37‐week aged, and 52‐week extremely old late‐life fish. Briefly, snap‐frozen muscle samples from comparable anterior–posterior positions were cryo‐pulverized and the metabolites were extracted and subject to liquid chromatography coupled to high‐resolution mass spectrometry (LC–MS) (Figure 4a). Using this approach, we putatively identified 1160 metabolites across the 20 samples. Principal component analysis (PCA) of all metabolites revealed clear clustering of the three time points, reinforcing the distinctness of the various life‐history phases (Figure 4b). Interestingly, the PCA scores plot showed that the 22‐week samples fell in between the 37‐week and 52‐week samples in the first principal component, and unsupervised hierarchical clustering analyses revealed that the 22‐week early life and 52‐week late‐life datasets are more closely related to each other than the 37‐week aged fish (Figure 4c). This suggests that some aspects of metabolism in the extremely old stage resembles an early life state. On the other hand, in the second principal component, the 37‐week aged, and 52‐week extremely old fish are more similar to each other, highlighting a different set of metabolites that are not reversed during late‐life in the way that those in the first principal component are. Overall, our global muscle metabolomics analyses suggest that some metabolic processes are reversed coincidental with the onset of the extremely old phase.

We next applied a systems biology approach to dissect, in an unbiased fashion, the specific changes occurring during the three different stages. For this purpose, we used analysis of variance (ANOVA) and Pearson correlation analysis to identify groups of metabolites that displayed similar or significant changes in metabolite abundance across the three time points. Using this strategy, each metabolite is categorized as uniquely belonging to one of 17 trends (Extended Data Figure 2 in Data S1), which are broadly categorized in five groups (Figure 4d): Metabolites within group 1 display a monotonic behavior in that their abundance does not significantly change throughout the life span of the fish (Figure 4d). Metabolites that significantly change with age can be placed in one of four groups corresponding to: upregulated with age, downregulated with age, a bell‐shaped inversion whereby their abundance at the 37‐week aged time point is higher than 22‐week early life and 52 extremely old week cohorts, or a U‐shaped inversion in that their abundance at 37‐week is lower than 22‐weeks and 52‐weeks (Figure 4d). Unsurprisingly, for the majority of metabolites (72.1%), no significant changes with age are observed (Figure 4e). However, of the metabolites that are significantly altered with age, the bell‐shaped inversion group was found to be the predominant group accounting for 12.8% of all metabolites (Figure 4e). The enrichment of metabolites within the bell‐shaped inversion group, characterized by increased abundance in the aged group and indistinguishable levels between the early life and extremely old muscle, supports our argument that late in life, specific aspects of metabolism revert to levels of activity that more closely resemble early life.

To identify specific metabolites that maybe central in distinguishing and/or driving aging and extremely old stages, we assessed the correlation of the levels of metabolites across the three sampling time points, following which an association network was constructed (Figure 4f). Remarkably, we found that metabolites that are most centrally located within the network, and most interconnected with other metabolites, predominantly belong to the bell‐shaped inversion group, reinforcing that the bell‐shaped metabolites may be the key players regulating metabolism in aged and extremely old animals. To identify the most influential metabolites within the network, we used our novel “Integrated Value of Significance” (IVI) algorithm (Salavaty et al., 2020), which combines different centrality measures to capture all topological dimensions of the network, subsequently ranking each metabolite based on the authoritative position it holds within the network (see methods section for detailed methodology). Metabolites with the highest IVI represents those that have the highest local and global influence in the entire network and based on this approach, the top 15 most influential metabolites are documented in Extended Data Table 1 in Data S1. Of these 15 metabolites, 12 display a bell‐shaped inversion trend, indicating that an accumulation of these metabolites during aging and subsequent reduction during the extremely old late‐life stages maybe central in distinguishing and physiologically regulating the two adult life‐history phases. Notably, 11 of these 15 metabolites correspond to lipids (Extended Data Table 1 in Data S1; Figure 4g), suggesting that lipid metabolism maybe the central pathway that regulates the entire metabolome, and drives the extension of life span.

### 2.6 Protein and lipid metabolism are overrepresented in the bell‐shaped group of metabolites

Since our analyses revealed that the bell‐shaped cluster was the largest group of significantly altered metabolites, and that this bell‐shaped abundance fits the expected profile of metabolites associated with the reduction in mortality at the late‐life stages, all further analyses were focused on this group. Using the approach described in the above section, we allocated each of the 149 metabolites exhibiting bell‐shaped inversion profiles to the respective pathways they are involved in. Our analyses revealed that with the exception of 14 metabolites, which were either absent from all databases or had no known function(s) in animals, all other metabolites could be categorized into one of eight major pathways (Figure 4h). Importantly, protein (29.5%) and lipid (28.2%) metabolites were found to be highly represented in the bell‐shaped group, making them the prime candidate pathways for the increased longevity of the extremely old group, and as such we examined them in further detail.

### 2.7 Increased protein breakdown is evident during aging

Since protein metabolism is one of the two highly represented pathways, we chose to examine it in further detail, specifically focusing on short (di‐ to tetra‐) peptides, which are known to be preferentially identified in our metabolomics workflow. We revealed that although 53 of the 85 peptides detected did not significantly change with age, more than a quarter exhibited a bell‐shaped inversion trend (Figure 4i). Peptides are commonly associated with proteolysis and protein turnover (Lecker & Goldberg, 2002). Since we have shown that during aging there is increased expression of the E3 ubiquitin ligase *atrogin1* (Figure 2a), which is known to breakdown proteins and promote atrophy, we argue that the protein metabolic changes we have identified are most likely a reflection of changes in proteolysis and the subsequent alterations in muscle fiber size. That is, increased *atrogin1*‐dependent protein catabolism during aging results in increased peptide abundance and muscle fiber atrophy. Conversely, reduced *atrogin1* expression in the extremely old late‐life stages results in reduced proteolysis and generation of peptide species subsequently stabilizing muscle fiber size. The changes in protein metabolism we have observed are therefore likely a reflection of changes in protein turnover, and subsequent alteration in muscle fiber size.

### 2.8 Lipid metabolism is altered in aged killifish

**FIGURE 5:**
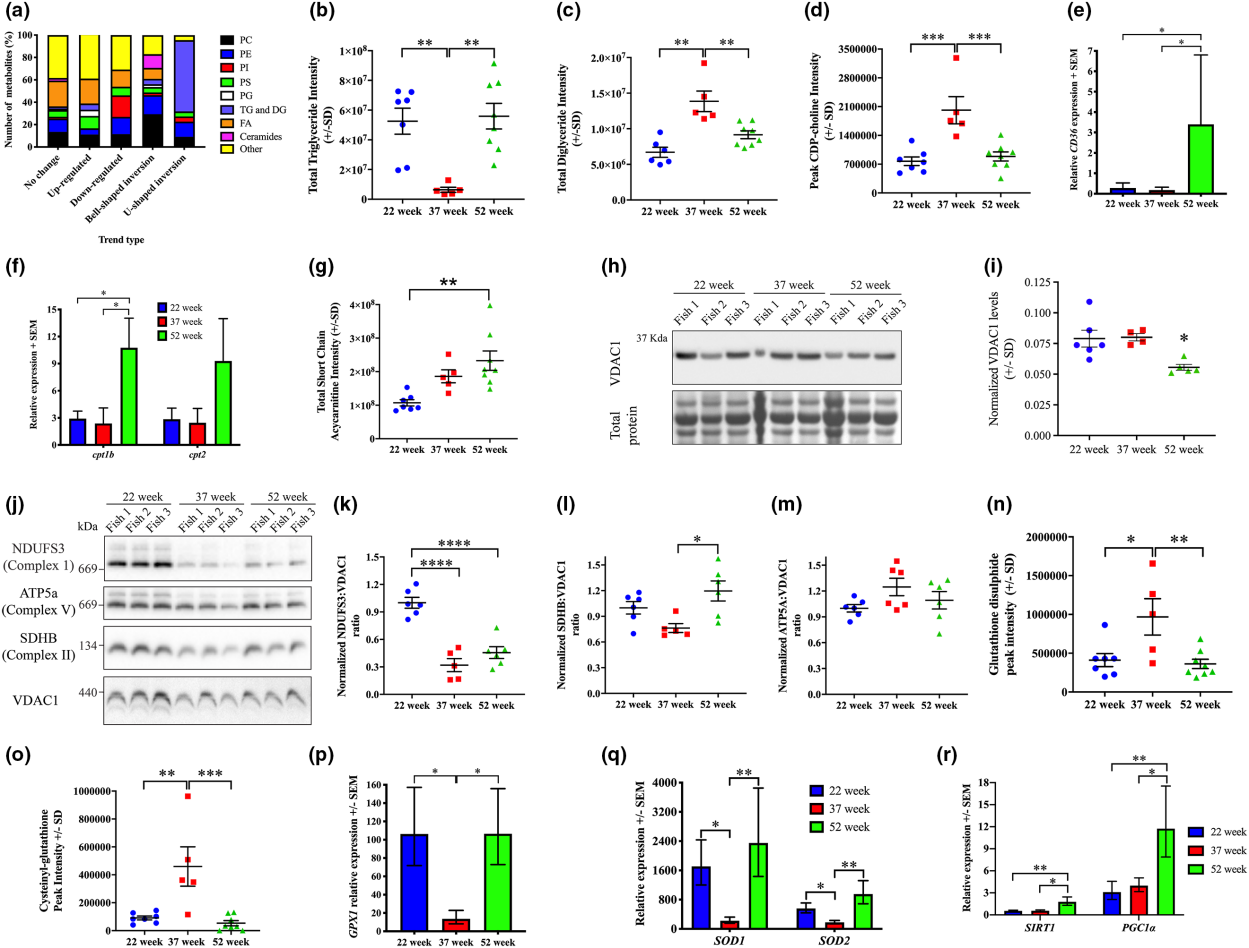
Depletion of triglycerides during aging results in increased reactive oxygen species which triggers mitohormesis in late‐life fish. (a) Distribution of all lipid metabolites across the five trend types: phosphatidylcholine (PC), phosphatidylethanolamine (PE), phosphatidylinositol (PI), phosphatidylserine (PS), phosphatidylglycerol (PG), triglycerides (TG), diglycerides (DG), and free fatty acids (FA). The “others” category consist of acylcarnitines, acylglycines, cardiolipins, sphingomyelins, and intermediates of lipid metabolism. Total intensity of triglycerides (b), diglycerides (c), and CDP‐choline (d). Error bars represent ± SD. (e) Gene expression level of *cluster of differentiation 36* (*CD36*) at each of the three timepoints. Error bars represent ± SEM. (f) Gene expression level of *carnitine palmitoyl transferase 1b* (*cpt1b*) and *carnitine palmitoyl transferase 2* (*cpt2*) at each of the three time points. Error bars represent ± SEM. (g) Total intensity of all short chain acylcarnitine species. Error bars represent ± SD. (h) Representative western blot images for VDAC1, and total protein direct blue stain, on whole cell protein lysates from 22‐week, 37‐week and 52‐week‐old male killifish. (i) Quantification of VDAC1 levels normalized to total protein. Error bars represent ± SD. (j) Representative BN‐PAGE blots for complex I protein NADH:Ubiquinone oxidoreductase core subunit S3 (NDUFS3), complex II protein Succinate dehydrogenase complex subunit B (SDHB), complex V protein ATP synthase F1 subunit alpha (ATP5a), and mitochondrial voltage‐dependent anion‐selective channel 1 (VDAC1) on mitochondrial lysates isolated from 22‐week, 37‐week, and 52‐week male killifish. (k–m) Intensities of NDUFS3, SDHB, and ATP5a normalized to VDAC1. Error bars represent ± SD. Total intensity of glutathione disulphide (*n*) and cysteinyl‐glutathione (o) in 22‐week, 37‐week, and 52‐week male killifish. (p‐q) Gene expression levels of antioxidant enzymes *glutathione peroxidase 1* (*GPX1*), cytosolic *copper‐zinc superoxide dismutase* (*SOD1*, *Cu*, and *Zn‐SOD*), and *manganese superoxide dismutase* (*SOD2*, *Mn‐SOD*). Error bars represent ± SEM. (r) Gene expression levels of *sirtuin 1* (*sirt1*) and *peroxisome proliferator‐activated receptor‐gamma coactivator 1 α* (*PGC1α*). Error bars represent ± SEM. *p < 0.05; **p < 0.01; ***p < 0.001; and ****p < 0.0001 calculated using one‐way ANOVA with Tukey's multiple correction post hoc test.

Given that lipid metabolism was the other pathway that was highly represented in the bell‐shaped inversion group (Figure 4h), and that it was also identified as the most influential pathway based on our IVI analyses (Figure 4h and Extended Data Table 1 in Data S1), we chose to examine it further. Examination of all lipid metabolites in our dataset revealed that multiple lipid subclasses are altered with age (Figure 5a). Of these, the most striking was triglycerides (TG) and diglycerides (DG), which are enriched within the U‐shaped inversion trend, the phosphatidylinositol (PI) phospholipids that are highly represented in the downregulated group, and ceramides which are overrepresented in the bell‐shaped inversion group (Figure 5a).

To better understand how lipid metabolism changes during ageing and extremely old late‐life stages, we determined the total abundance of each lipid type by adding the peak intensities of all significantly altered metabolites within that subtype. Using this approach, our analyses revealed significant changes in various phospholipids including the two most abundant species phosphatidylcholine (PC) and phosphatidylethanolamine (PE) (Extended Data Figure 3a,b in Data S1); and significant changes in the less abundant phospholipids phosphatidylinositol (PI), phosphatidylserine (PS), and phosphatidylglycerol (PG; Extended Data Figure 3c–e in Data S1). An accumulation of ceramides in 37‐week aged cohort was also evident (Extended Data Figure 3f in Data S1). Phospholipids and ceramides have critical roles in regulating muscle contraction (Funai et al., 2013, 2015), metabolism (Lee et al., 2018; Newsom et al., 2016), and apoptosis (Turpin et al., 2006) and as such, changes in their abundance during aging and late‐life may significantly impact muscle heath at the two stages, subsequently contributing to a reduction in mortality.

### 2.9 Triglyceride reserves are depleted in aged but not extremely old killifish

The lipid subclass that is most dramatically affected in our dataset is TG, which serves as an essential energy reserve in skeletal muscle (Figure 5a). We observed a typical U‐shaped trend with respect to TG levels in that they significantly dropped at 37‐weeks and accumulated at 52‐weeks (Figure 5a,b). We hypothesized that the reduction in TG at 37‐weeks is due to increased TG breakdown, and that at 52‐weeks, this process is inhibited, resulting in TG accumulation. In support of this hypothesis, we observed a significant increase in DG at 37‐weeks and a reciprocal reduction at 52‐weeks (Figure 5c). We also observed changes in the activity of the CDP‐choline pathway, which utilizes DG to synthesize the majority of PC pools. Consistent with the high levels of lipolysis evident at 37‐weeks, we observed a significant increase in the PC precursor CDP‐choline (Figure 5d), and in PC levels (Extended Data Figure 3a in Data S1) at 37‐weeks highlighting increased PC synthesis during aging. On the other hand, at 52‐weeks, both, CDP‐choline and PC levels were significantly reduced (Figure 5d; Extended Data Figure 3a in Data S1) suggesting that PC synthesis is downregulated during late‐life, concomitant with a reduction in the utilization of DG, resulting in reduced lipolysis and TG accumulation.

An alternative explanation for the changes in TG reserves is alterations in fatty acid uptake and/or TG disposal via fatty acid oxidation and oxidative phosphorylation (OXPHOS). To test the fatty acid uptake hypothesis, we examined the expression of *cluster of differentiation 36* (CD36; also known as fatty acid translocase), an integral membrane protein which transports fatty acids into the cell. Our analyses revealed that while the levels of *CD36* were unaffected at 37‐weeks, a significant increase in *CD36* expression was evident in 52‐week fish (Figure 5e). This result highlights that in aged killifish, there is no change in fatty acid uptake, consistent with the reduced TG reserves, but in the extremely old phase, there is increased fatty acid uptake, which may also contribute to the increased abundance of TG.

To test the fatty acid disposal hypothesis, we first examined fiber type distribution as a shift from glycolytic, type II fast muscle fibers to an oxidative, type I slow phenotype (Larsson et al., 1978; Lee et al., 2006), is routinely seen during sarcopenia, and can result in increased fatty acid oxidation and account for the reduction in TG reserves. Using a myosin antibody that detects both slow and fast isoforms, and one that specifically detects the slow isoform, we reveal that in all stages, the slow muscle, stained with both myosin antibodies, is superficially located, with the fast muscle fibers, which is stained by a single myosin antibody, occupies the rest of the myotome (Extended Data Figure 4a–c in Data S1). Importantly, we did not see any slow muscle fibers deeper in the myotome highlighting the absence of fiber type switching (Extended Data Figure 4a–c in Data S1). We also examined protein levels of Myosin 7, which is the slow muscle isoform, and of myosin light chain 2/3, which is exclusively found in fast muscles (Extended Data Figure 4d in Data S1). We observed no difference in the abundance of either of the two protein isoforms, confirming that fiber type switching and a shift to fatty acid oxidation is not a feature of muscle ageing in killifish (Extended Data Figure 4e in Data S1). This result is further supported by our metabolomics data, which revealed that metabolites involved in glycolysis and carbohydrate metabolism are unaffected during the life span of the fish (Extended Data Table 2 in Data S1). Our results therefore suggest that the reduction in TG at 37 weeks is not due to a switch in the preference of substrate utilization for the production of energy.

To further explore if changes in fatty acid oxidation and oxidative phosphorylation (OXPHOS) are responsible for the dramatic differences in TG abundance, we examined the expression of the enzymes *carnitine palmitoyl transferase 1b* (*cpt1b*) and *carnitine palmitoyl transferase 2* (*cpt2*), two enzymes known to transport activated fatty acids, in the form of acylcarnitines, across the outer and inner mitochondrial membranes, respectively. Our results indicate that both, *cpt1b* and *cpt2* levels remained unaltered in the 37‐week samples (Figure 5f), suggesting that the transport of activated fatty acids across the mitochondrial membrane is not altered during aging. At 52‐weeks, however, we observed a significant increase in the expression of *cpt1b* (Figure 5f), demonstrating that during late‐life there may be an increase in translocation of activated fatty acids into the mitochondria. We also examined how acylcarnitine species are affected during the life span of the fish. Although a combination of short (C2–C6), medium (C7–C12), and long chain (C13–C22) acylcarnitine species were identified in our metabolomics dataset, only short chain acylcarnitine species were found to be significantly affected, as identified by our ANOVA analyses (Extended Data Figure 3g in Data S1). Examination of the total abundance of all short chain acylcarnitine species revealed a significant increase in 52‐week fish but not the 37‐week group, further supporting increased fatty acid oxidation during late‐life, but not aging (Figure 5g). As further validation for this argument, we examined total mitochondrial content, using western blot for VDAC1 on whole cell protein lysate, and assessed the abundance of various mitochondrial OXPHOS complexes in isolated mitochondria, using blue native gel electrophoresis (BNE). While VDAC1 levels in 37‐week fish was unaffected, we observed a significant reduction in the 52‐week time point (Figure 5h,i), indicating a reduction in mitochondrial content during the extremely old late‐life phase. Additionally, examination of the abundance of OXPHOS complexes revealed a decrease in Complex I in the 37‐week aged and 52‐week late‐life cohorts, a mild increase in Complex II at 52‐weeks but no changes in the abundance in Complex V (Figure 4j–m). These results suggest that mitochondria number, and function decline with increasing age, supporting the argument that fatty acid oxidation is unlikely to be responsible for the lower levels of TG displayed.

Therefore, collectively our results highlight that while during aging, there is a reduction in TG pools, likely due to increased lipolysis, in the extremely old cohort, there is reduced lipolysis, and an increase in fatty acid uptake and fatty acid oxidation, the net effect of which is TG accumulation.

### 2.10 Reactive oxygen species‐mediated mitohormesis is triggered in late‐life

The striking depletion of triglycerides observed in 37‐week aged fish resembles calorie/dietary restriction in that they are both characterized by a reduction in nutrient availability. Calorie restriction in particular, has been shown to extend life span in a multitude of organisms, by transiently increasing reactive oxygen species (ROS) and subsequently triggering a protective stress response—termed mitohormesis, which alters the transcriptional state of the cell thus increasing stress resistance and improving cellular metabolism (Agarwal et al., 2005; Cox et al., 2018; Mesquita et al., 2010; Owusu‐Ansah et al., 2013; Schulz et al., 2007; Sharma et al., 2010; Weimer et al., 2014; Zarse et al., 2012). Additionally, a reduction in OXPHOS complexes following genetic and/or pharmacological manipulations has also been shown to trigger ROS‐mediated mitohormesis and extend life span in multiple organisms including killifish (Baumgart et al., 2016; Copeland et al., 2009; Durieux et al., 2011; Feng et al., 2001; Lee et al., 2002). Given that aged killifish display both, triglyceride depletion resembling a state of calorie restriction, and show a dramatic reduction in OXPHOS complexes, we hypothesized that ROS‐mediated mitohormesis may be activated in late‐life fish culminating to their increased life span and overall reduction in mortality rates. To test this hypothesis, we examined the redox status of skeletal muscle at the various time points. We first examined the levels of glutathione, an important scavenger of ROS, in the metabolomics data. While the abundance of glutathione is unaltered (Extended Data Figure 3h in Data S1), we observed a significant increase in its oxidized form glutathione disulphide (Figure 5n), and another oxidized disulphide, cysteinyl‐glutathione (Figure 5o), at 37‐weeks, pointing to increased abundance of ROS. Given the high variability observed for these glutathione metabolites, likely due to the propensity for thiols to readily oxidize in the samples, we also examined the levels of the antioxidant enzymes *glutathione peroxidase 1* (*GPX1*), *cytosolic copper‐zinc superoxide dismutase* (*SOD1*, *Cu‐Zn‐SOD*), and *manganese superoxide dismutase* (*SOD2*, *Mn‐SOD*). Our analyses reveal a striking reduction in *GPX1*, *SOD1*, and *SOD2* in the 37‐week aged fish (Figure 5p,q), and this may further increase ROS, which subsequently activates mitohormesis.

Remarkably, although the antioxidant genes *GPX1*, *SOD1*, and *SOD2* were significantly reduced in aged killifish, in the extremely old stage, their expression was comparable to early life cohorts (Figure 5p,q), supporting the activation of mitohormesis in late‐life fish. Since calorie restriction and ROS have been shown to trigger mitohormesis by upregulating the sirtuin signaling pathway (Chen et al., 2005; Cohen et al., 2004; Heidler et al., 2009; Schmeisser et al., 2013) (reviewed in [Ristow & Schmeisser, 2014]), we examined the levels of *sirtuin 1* (*sirt1*). Indeed, we observed a significant increase in *sirt1* expression in 52‐week late‐life fish compared to 22‐week early life and 37‐week aged cohorts (Figure 5r). Finally, we also observed a significant increase in the transcriptional coactivator *peroxisome proliferator‐activated receptor‐gamma coactivator 1‐α* (*PGC1α*) (Figure 5r), a known target of Sirt1 that regulates mitochondrial biogenesis and metabolism. Collectively, these results support a role of ROS‐mediated mitohormesis and upregulation of Sirt1 in increasing longevity of the extremely old, late‐life cohort.

### 2.11 Lipid droplet distribution is altered in extremely old late‐life killifish

**FIGURE 6:**
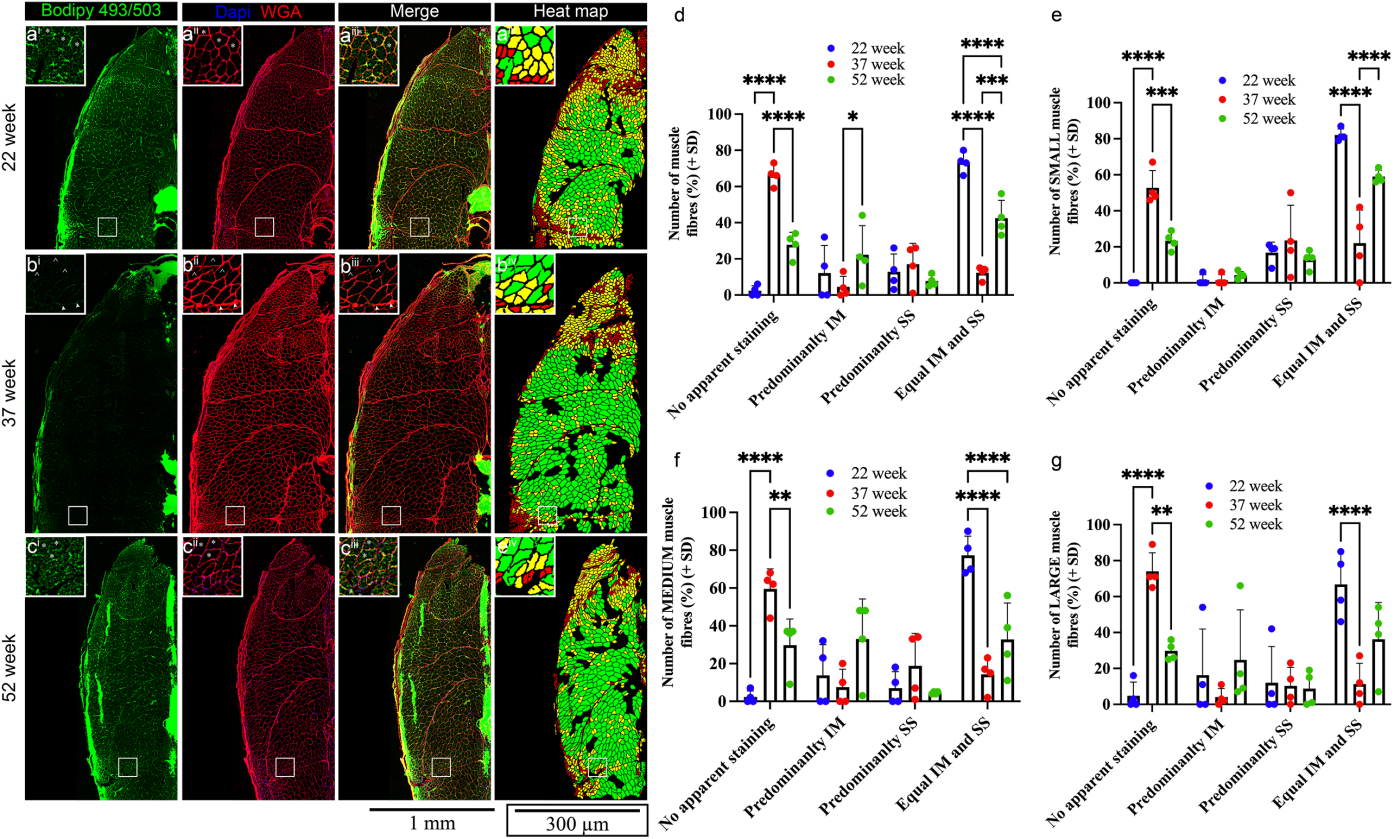
Lipid droplet distribution is altered in extremely old late‐life killifish. (a–c) Representative images of intracellular lipid droplets, labelled with Bodipy (493/503) (green), Dapi (blue), and wheat germ agglutinin (WGA) (red) in 22‐week, 37‐week, and 52‐week muscle. Heat map of fiber size distribution for each animal also displayed. Zoom in views highlight the different lipid pools: no apparent Bodipy (493/503) staining (^), predominantly subsarcolemmal (SS) staining (arrowhead), predominantly intermyofibrillar (IM; #), or equal distribution of SS and IM localization (*). (d) Percentage of total muscle fibers displaying no apparent Bodipy staining, predominantly SS, predominantly IM, or equal distribution of SS and IM localization. Error bars represent ± SD. (e) Percentage of small muscle fibers displaying no apparent Bodipy staining, predominantly SS, predominantly IM, or equal distribution of SS and IM localization. Error bars represent ± SD. (f) Percentage of medium muscle fibers displaying no apparent Bodipy staining, predominantly SS, predominantly IM, or equal distribution of SS and IM localization. Error bars represent ± SD. (g) Percentage of total muscle fibers displaying no apparent Bodipy staining, predominantly SS, predominantly IM, or equal distribution of SS and IM localization. Error bars represent ± SD. *p < 0.05; **p < 0.01; and ****p < 0.0001 calculated using two‐way ANOVA with Tukey's multiple correction post hoc test.

While high levels of intracellular lipid content is often associated with metabolic pathologies such as type 2 diabetes mellitus and obesity (Bonen et al., 2000; Jacob et al., 1999; Pan et al., 1997), the accumulation of TG in athletes and endurance trainers has been shown to improve insulin sensitivity (Goodpaster et al., 2001; Jacob et al., 1999; Schenk & Horowitz, 2007), a phenomenon known as the athlete's paradox. The different effects of TG accumulation is partly mediated through differences in lipid droplet dynamics, with diabetic patients accumulating lipids in large subsarcolemmal (SS) lipid droplets, and lipid stores in athletes' enriched within smaller but more abundant intermyofibrillar (IM) droplets (Daemen et al., 2018). Given that TG accumulation following exercise has been shown to be mediated by *PGC1α* (Koves et al., 2012), which is also upregulated in the muscle from extremely old killifish, we hypothesized that they may display a similar lipid droplet localization to athletes, and this may be a key factor preventing metabolic pathologies and mortality, and instead promote their survival. To assess this, we used the Bodipy (493/503) dye to visualize the distribution of lipid droplets in 22‐, 37‐, and 52‐week muscle samples (Figure 6a–c). Consistent with our hypothesis, at 22‐weeks, the majority of fibers displayed equal distribution of SS and IM localized droplets, with very few showing no apparent staining (Figure 6a,d). In stark contrast, at 37‐weeks, the majority of fibers displayed no apparent Bodipy staining (Figure 6b,d). Interestingly, despite the increase in TG levels in 52‐week late‐life samples, approximately 30% of fibers were scored as having no apparent staining (Figure 6c,d). This suggests that the increase in TG reserves during late‐life stages is not homogenous, and some fibers accumulate TG more than others. However, of the fibers that display TG accumulation, we observed a striking increase in the number of cells displaying IM localization and those displaying equal distribution of SS and IM at the 52‐week stage compared to 37‐week aged fish (Figure 6c,d). We further evaluated if the changes in lipid distribution observed were associated with the size of the fiber. To do this, we combined our fiber size heat map strategy with Bodipy staining and scored the lipid distribution in 20–30 muscle cells from each of three size categories. In line with the above analyses on all muscle cells, the majority of small (Figure 6e), medium (Figure 6f), and large (Figure 6g) fibers showed equal distribution of SS and IM localized droplets at 22‐weeks. However, at 37‐weeks, each of the three fiber sizes displayed an increase in the number of cells with no apparent staining, and a reciprocal reduction in the cells showing equal distribution of SS and IM localized droplets. Finally, at 52‐weeks, there was a significant reduction in small, medium, and large cells showing no apparent staining, although only the small fibers displayed a significant increase in SS and IM localized droplets. Collectively, these results highlight that TG reserves not only increase in extremely old animals, but there is also a shift in the distribution of TG droplets such that there is an increase in IM, and equally distributed SS and IM localized pools. This distribution is highly similar to that of endurance‐trained athletes and has been thought to provide a high surface area to volume ratio for more efficient utilization for energy.

### 2.12 Short‐term resveratrol treatment and subsequent Sirt1 upregulation in aged killifish results in a late‐life like phenotype

**FIGURE 7:**
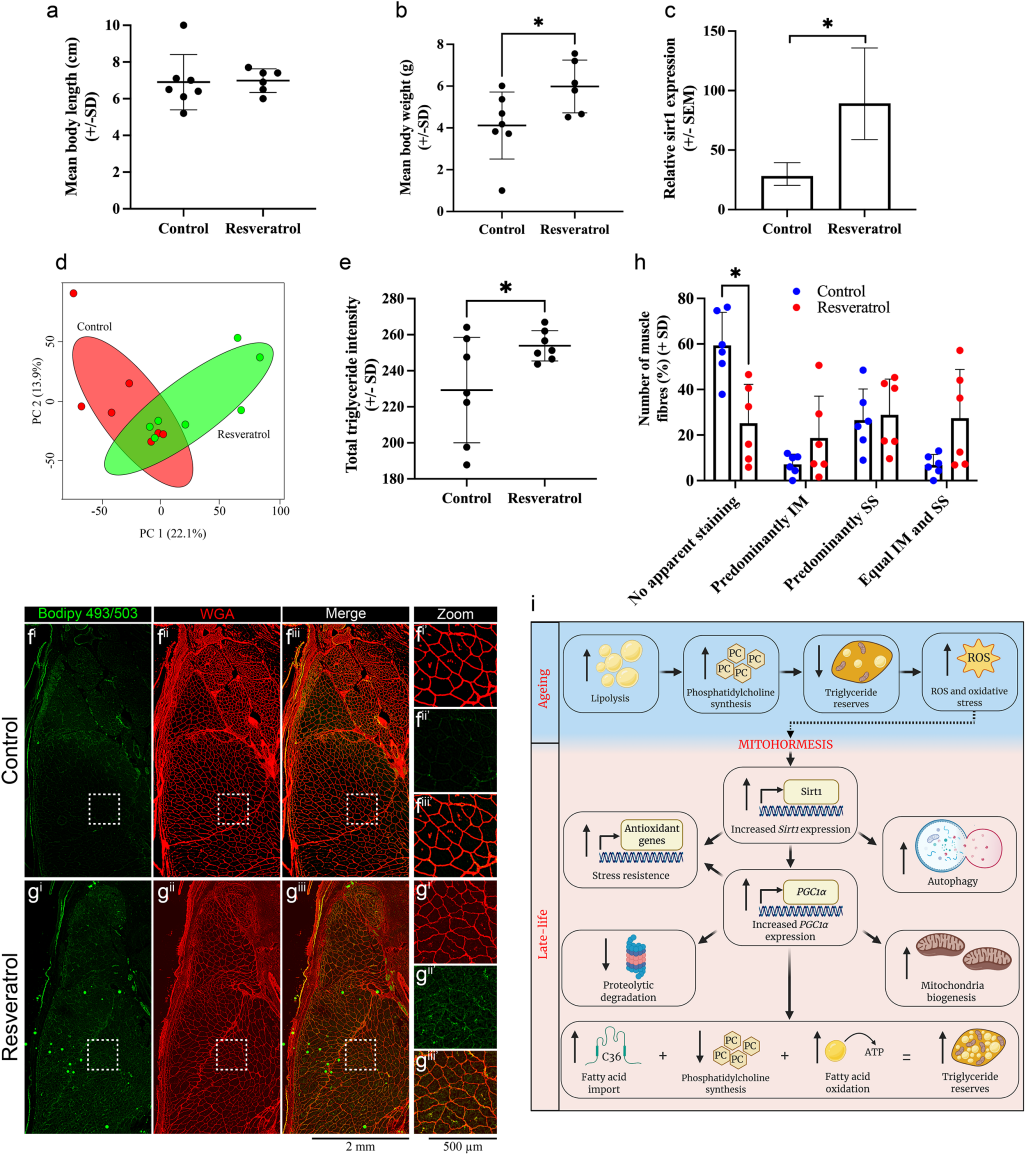
Short‐term resveratrol treatment and subsequent *sirt1* upregulation in aged killifish results in a late life‐like phenotype. Quantification of body length (a) and weight (b) of control and resveratrol‐treated 37‐week aged fish. Error bars represent ± SD. (c) Gene expression levels of *sirt1* in control and resveratrol‐treated 37 week aged fish. Error bars represent ± SEM. (d) Principal component analyses score plot of all metabolites identified in control (red) and resveratrol (green)‐treated muscle lysates. (e) Total intensity of triglycerides (TG) in control and resveratrol‐treated animals. Error bars represent ± SD. (f–g) Representative images of intracellular lipid droplets, labelled with Bodipy (493/503) (green) and wheat germ agglutinin (WGA) (red) in control and resveratrol‐treated fish. (h) Percentage of total muscle fibers displaying no apparent Bodipy staining, predominantly SS, predominantly IM, or equal distribution of SS and IM localization in control and resveratrol‐treated muscle. Error bars represent ± SD. *p < 0.05 calculated using unpaired *t* test (one‐tailed) (a–e) or Unpaired *t* test with Holm–Šídák method multiple comparison (h). (i) Schematic of the proposed mechanisms driving mortality deceleration. Created with BioRender.com.

Based on our results thus far, we propose the following model for increased longevity of the extremely old animals: During aging, the depletion of triglycerides increases ROS and oxidative stress, which triggers mitohormesis. As a result, there is a significant increase in Sirt1 and *PGC1 α* expression during late life, which not only increases the expression of antioxidant genes thus reducing oxidative stress, but also improves lipid metabolism, including more efficient distribution of lipid droplets. This model implicates Sirt1 upregulation in the maintenance of nutrient homeostasis and the deceleration of mortality rates. To functionally validate this model, we treated 33‐week old killifish with the well‐characterized Sirt1 inducer resveratrol (Ma et al., 2020; Nishigaki et al., 2020; Yang et al., 2019), for 4‐weeks, and at 37‐weeks, we examined their metabolome focusing on lipid metabolism. We hypothesized that resveratrol treatment would result in increased Sirt1 expression in an aged animal, which according to our model is sufficient to promote a late‐life phenotype in the treated animals. Examination of body size revealed no change in the length, but a significant increase of body weight of resveratrol treated males compared to control vehicle‐treated males (Figure 7a,b). Importantly, the resveratrol treatment regime used was sufficient in inducing a significant increase in *Sirt1* mRNA levels (Figure 7c).

Having confirmed the induction of *sirt1* levels, we performed an untargeted metabolomics comparing the metabolome of resveratrol treated fish to that of control animals, identifying 1398 putative metabolites across 14 samples (seven control and seven resveratrol‐treated animals). PCA plot of all metabolites revealed clear clustering of the control and resveratrol‐treated groups, highlighting their distinct metabolic states (Figure 7d). This was particularly remarkable given the short 4‐week treatment regime used. Given that lipid metabolism was the most striking difference observed between aging and late‐life stages, we next examined how it was altered following resveratrol treatment. As per previous analyses, we determined the total abundance of each lipid type by adding the peak intensities of all significantly altered metabolites within that subtype. Consistent with our model, our analyses revealed a significant increase in triglyceride levels in resveratrol‐treated males compared to control animals (Figure 7e). While we observed no differences in DG levels (Extended Data Figure 5a in Data S1), we saw a small, near‐significant reduction in CDP choline suggesting that similar to late‐life stages (Extended Data Figure 5b in Data S1), resveratrol‐treated animals display reduced PC synthesis, which may result in reduced lipolysis and subsequent TG accumulation. Furthermore, various other lipid subclasses were altered in resveratrol animals. This includes a significant reduction in PC (Extended Data Figure 5c in Data S1), PE (Extended Data Figure 5d in Data S1), and PS levels (Extended Data Figure 5f in Data S1), all of which also reduce during late‐life, and increased PG levels (Extended Data Figure 5g in Data S1), although PI (Extended Data Figure 5e in Data S1) and ceramides remain unaffected (Extended Data Figure 5h in Data S1). Collectively, these results highlight striking similarities in lipid composition between resveratrol‐treated and late‐life stage animals thus providing support for our model.

We next examined if, in addition to increased TG levels, the lipid distribution was also altered in the resveratrol‐treated animals. For this purpose, we scored the lipid droplet localization in control and resveratrol‐treated muscle fibers, into one of the four categories: no apparent staining, predominantly IM, predominantly SS, and equal IM and DD distribution. Consistent with the changes seen in late‐life, we observed a significant reduction in the number of fibers displaying no apparent staining in the resveratrol‐treated animals (compared to control), and this was accompanied with small, nonsignificant increases in predominantly IM and equal IM and SS fibers (Figure 7f–h). Collectively, this data provides functional evidence on the role of Sirt1 in regulating lipid metabolism during late life, thus supporting our model on the role of mitohormesis in regulating mortality deceleration.

## 3 DISCUSSION

In this study, we have demonstrated that killifish display many of the hallmarks of sarcopenia—including muscle fiber atrophy, altered proteolysis, reduced stem cell stem number, and denervation—thus providing an extremely short‐lived vertebrate model system to study the biology of sarcopenia with high repeatability and feasibility.

Through our extensive characterization experiments, we also uncovered that while many of these features deteriorate with increasing age, a small number of features—fiber size and proteolysis—improved in late‐life cohorts, coinciding with when mortality rates decline. This suggests that in extremely old animals, there may be mechanisms in place that prevent further deterioration of skeletal muscle health, ultimately contributing to an extension of their life span. Indeed, it has been reported in numerous organisms that while aging is initially characterized by an exponential increase in mortality, at later stages of adult life, mortality rates either plateau or even decline, a phenomenon termed mortality deceleration (Barbi et al., 2018; Carey et al., 1992; Chen et al., 2013; Curtsinger et al., 1992; Shahrestani et al., 2009; Vaupel et al., 1998). The existence of mortality deceleration is intriguing because it suggests that beyond a certain time point, aging may cease, or even be reversed. Our findings that mortality rates decline, at a population level, in the late‐life stages demonstrates that killifish also undergo mortality deceleration. Killifish therefore provide a novel model to study the mechanistic basis of this phenomenon in a vertebrate, with the metabolomics analyses presented here‐in, detailing the metabolic status of skeletal muscle from each of the three time points, being the first attempt toward this goal. Our analyses have revealed a central role of lipid metabolism in regulating the deceleration of mortality rates in the extremely old, late‐life stages. Based on our observations and previously published literature, we propose the following model for the increased longevity of extremely old animals (Figure 7i): During aging, there is a depletion of TG reserves, resulting in the reduced availability of nutrients and energy, which triggers increased ROS production, a response that is commonly seen following calorie restriction regimes. This, combined with the reduction in mitochondrial OXPHOS complexes activates mitohormesis in a small number of fish, resulting in increased s*irt1* expression, which mediates multiple downstream molecular changes subsequently extending life span. Indeed, we have shown that upregulation of s*irt1* following treatment with resveratrol was sufficient to induce a late life‐like metabolic profile in aged animals, supporting its role in the deceleration of mortality. Further to this, both, resveratrol (Valenzano et al., 2006) and dietary restriction (Terzibasi et al., 2009) have been shown to not only reduce age‐related pathologies in killifish, including improved learning and memory, reduced neurodegeneration, and reduced lipofuscin accumulation, but also prolong maximum life span. Collectively, these results highlight a role of Sirt1 in regulating the metabolic alterations seen in late‐life stages.

Based on published literature, increased Sirt1 has numerous downstream effects, many of which were also evident in the extremely old cohort (Figure 7i). These include: increased expression of antioxidant genes, which reduces oxidative stress during late life (Pardo et al., 2010); autophagy induction, which reduces muscle atrophy and promotes longevity (Hansen et al., 2018); and increased expression of *PGC1α* (Rodgers et al., 2005), the master regulator of lipid and mitochondrial metabolism. The induction of *PGC1α* has also been shown to have multiple downstream effects: increased expression of antioxidant genes, including *SOD* and *GPX*, thus contributing to reduced oxidative stress during late life (St‐Pierre et al., 2006); Inhibition of ubiquitin ligase expression, including *atrogin1*, subsequently reducing the proteolytic degradation of proteins, and thus preventing further muscle atrophy during the late‐life phase (Brault et al., 2010; Sandri et al., 2006); and altered lipid metabolism (Gerhart‐Hines et al., 2007; Wu et al., 1999), the net effect of which is accumulation of TG in the late‐life cohort. Importantly, intracellular lipids are stored within smaller, more abundant intermyofibrillar (IM) droplets, which has been shown to improve the utilization for energy, and it is possible that the same occurs in late‐life stages, subsequently resulting in the maintenance of nutrient and energy homeostasis. We, therefore, argue that although a high level of intracellular lipid content is often associated with metabolic pathologies, the accumulation of TG in late‐life muscle may be a key factor preventing metabolic pathologies and subsequent mortality and promoting survival. Indeed, wild‐type mice who mobilize fats less efficiently have been shown to live longer (Liao et al., 2011). Additionally, increasing specific types of dietary fats have also been shown to extend life span, and improve mitochondrial dynamics in aged skeletal muscle (Gutiérrez‐Casado et al., 2018), highlighting that the maintenance of adiposity can drive longevity.

The identification that mitohormesis and Sirtuin signaling are active at stages when mortality rates decrease is an exciting finding. Indeed, ROS and mitohormesis following pharmacological and/or genetic manipulations has been shown to extend longevity in various vertebrate models (Cox et al., 2018; Ost et al., 2014; Weimer et al., 2014), supporting its role in regulating the extremely old, late‐life stage. Additionally, overexpression of Sirtuin orthologs has also been shown to extend longevity in many species (Kaeberlein et al., 1999; Mouchiroud et al., 2013; Rogina & Helfand, 2004; Schmeisser et al., 2013; Tissenbaum & Guarente, 2001). However, our findings indicate the occurrence of mitohormesis in vertebrates that naturally live longer and suggest a role for it in life span extension in naturally occurring vertebrate populations. Therefore, an increased ability to respond to stress maybe a major evolutionary determinant of longevity and underpin the existence of late–life mortality deceleration.

In conclusion, we propose that ROS‐dependent mitohormesis and the upregulation of Sirt1 and *PGC1α* improves muscle health span during aging, providing a mechanism for the increased longevity in extremely old animals. Importantly, our results highlight that some of the metabolic hallmarks of aging can cease beyond a certain point, which is an exciting prospect given the social, economic, and health‐care costs associated with the ever‐growing aged population around the globe. The killifish model, therefore, not only provides an extremely short‐lived vertebrate to study the biological processes that govern aging, including sarcopenia, but also provides a unique model to understand the regulation of longevity and processes that control mortality deceleration.

## 4 METHODS

### 4.1 Experimental model and subject details

The *Nothobranchius furzeri* strain MZCS_08/122 was used for all experiments described herein, and the fish are originally derived from research‐purpose breeder (2010/63/EU). To avoid any confounding effects of egg production, which is known to impact metabolism, only male fish were examined at each of the four stages (6‐week, 22‐week, 37‐week, and 52‐week). Fish were kept at 26°C on a 12 h light: 12 h dark cycle. Fish are differentially fed according to their age: From hatching (0 days post fertilization) until 3–4 weeks posthatching, animals are fed with *Artemia salina* twice per day; juveniles (from 4 to 12 weeks) are fed with *Chironomidae spp*. larvae once per day and *Artemia salina* twice per day; and adults (from 12 weeks onward) are fed with *Chironomidae spp* larvae once per day. Animals are either group‐housed in conventional 20 L tanks or single‐housed in 5 L tanks (separated in two: 2.5 L per fish), such that the maximum number of fish per liter is 1. The health of the fish, including feeding behaviors, was monitored on a daily basis, and no viral or parasitic infections were evident in the fish utilized in this study. All animals were maintained in accordance with the current version of the German Law on the Protection of Animals.

### 4.2 Tissue collection

Fish were killed by incubating in 1 g/L tricaine methanesulphonate solution for 10 min. To avoid effects of circadian rhythms and feeding, animals were always culled between 10 am and 12 pm in a fasted state. The length and weight of each fish was measured following which, the muscle was dissected and either fixed in 4% paraformaldehyde (4% PFA), and subsequently embedded in paraffin or OCT medium, or snap‐frozen by immersing into liquid nitrogen.

### 4.3 Immunofluorescence staining

OCT‐embedded muscle was sectioned using a Leica CM 1850 cryostat and the sections were placed on poly‐lysine‐coated slides, which were stored at −80 until ready for staining. The sections were thawed by placing at room temperature for 30 min, fixed with 4% PFA for 5 min, and subsequently washed three times in phosphate‐buffered saline (PBST). The sections were then incubated in blocking media for 30 min, followed by overnight incubation in blocking solution containing appropriate antibody. Primary antibodies used in this study include: anti‐collagen 1 (Abcam, ab23730, 1/300), anti‐pax7 (Santa Cruz, sc‐81,648, 1:100), anti‐synaptic vesicle glycoprotein 2A (SV2, DSHB, 1/10), anti‐myosin (labelling slow and fast Myosin, A4.1025, DSHB, 1/10), and anti‐slow myosin (F59, DSHB, 1/10). The primary antibody was washed at least six times with PBST, following which the slides were incubated in secondary antibodies for 2 h. Secondary antibodies used are as follows: AlexaFluor™‐labeled‐488, AlexaFluor™‐labelled‐546, and/or AlexaFluor™‐labeled‐596 (Invitrogen, 1:300). Dapi (Sigma, D8417, final concentration 3 μg/mL) was used to counterstain nuclei, 594 conjugated α‐bungarotoxin dye (Biotium, 00007, final concentration 2 μg/mL) was used to label nicotinic acetylcholine receptors, Bodipy (493/503) (ThermoFisher Scientific, D3922, final concentration 10 μg/mL) for lipid droplets and conjugated wheat germ agglutinin (Vector Labs; RL‐1022 1:300 or Invitrogen, W32466, final concentration 5 μg/mL) to label the muscle membranes. Stained sections were mounted in SlowFade (ThermoFisher Scientific, S36963) mounting media or 80% glycerol and subsequently imaged using the Zeiss LSM 710 confocal microscope (SV2 and α‐bungarotoxin‐stained sections), the Zeiss Z1 compound microscope (collagen‐stained sections), the Leica DMI AF6000LX (A4.1025 and F59‐stained sections), or the Leica Thunder Deconvolution microscope (Pax7/WGA/Dapi‐stained sections and Bodipy/WGA (493/503)‐stained sections). Data analysis and maximum intensity projections (where appropriate) were obtained using Fiji (http://fiji.sc).

### 4.4 Protein isolation and western blot experiments

Protein lysates were obtained as per Boglev et al., 2013; Ruparelia et al., 2016 and quantified using the Qubit fluorometric quantification (Thermo Fisher Scientific). A total quantity of 20–30 μg of each sample, along with reducing agent (Life Technologies) and protein loading dye (Life Technologies), was heated at 70°C for 10 min, separated by SDS‐PAGE on NuPAGE 4–12% Bis‐Tris gels (for LC3, p62 and F310 staining) or NuPAGE Tris acetate gels (for F59 staining), and transferred onto PVDF membrane (Millipore). Following transfer, the membrane was blocked with 5% skimmed milk in PBST or TBST and subsequently probed with one of the following primary antibodies: anti‐LC3 (Cell Signaling, 12,741, 1/2000), anti‐p62 (Cell Signaling, 51,142, 1/1000), anti‐VDAC1, (Abcam, ab154856, 1/1000), anti‐Slow Myosin Heavy Chain (DSHB, A4/1025, 1/10), and anti‐Myosin light chain 1/3 (DSHB, F310, 1/10). The following morning, the primary antibody was washed and the membrane was incubated in the appropriate HRP‐conjugated secondary antibody (1:10000, Southern Biotech). Immunoblots were developed using ECL prime (GE healthcare) and imaged using a chemiluminescence detector (Vilber Lourmat). The membrane was subsequently stripped by incubating in 1X stripping buffer (200 mM glycine, 0.1% SDS, 1%Tween20, and pH 2.2) twice for 10 min, washed in PBST, and stained with Direct blue as per Zeng et al. (2013) to detect total protein. The blot images were quantified using Image Lab software (Bio‐Rad) and a one‐way ANOVA statistical test was used to test for significant changes in protein levels.

### 4.5 RNA extraction, cDNA synthesis, and qRT‐PCR

Snap‐frozen skeletal muscle was cryogenically pulverized (cryopulverization) using a 12‐well biopulverizer (BioSpec Products, OK USA Part number 59012MS) according to the manufacturer's instructions. Briefly, the biopulverizer and pestles were cooled by immersing in liquid nitrogen. Frozen samples were then added to a well and pulverized by striking the pestle four or five sharp blows with a mallet. The powdered tissue was then transferred using a spatula that had been cooled in liquid nitrogen into a cold Eppendorf tube on dry ice. Total RNA was extracted from the powdered tissue using TRI Reagent (Sigma, 93,289), and cDNA was subsequently synthesized using the iScript cDNA synthesis kit (Bio‐Rad, 1,708,891). Quantitative RT‐PCR (qRT‐PCR) was performed using a Mic qPCR cycler (Bio Molecular Systems) using SYBR Green Master mix (Life Technologies Australia, 4,364,346). All primers were tested to ensure a single product of 150–200 bp was amplified. Sequences of all primers used are listed in Extended Data Table 3 in Data S1.

### 4.6 Modelling of life span dataset

Using the survival data from our previously published life span analyses (Wendler et al., 2015), we calculated the cumulative mortality, at each week, which was then transformed on a logarithmic scale. The inflection points of the log‐scaled mortality data for males and females were assessed based on the extremum surface estimator method using the inflection R package (https://cran.r‐project.org/package=inflection). This method works based on the Lemmas defined in (Christopoulos, 2016).

### 4.7 Metabolomics experiments

#### 4.7.1 Sample preparation

Samples were randomized and blinded prior to preparation for metabolomic analysis. Snap‐frozen muscle was cryogenically pulverized as discussed above. The frozen tissue was then weighed by transferring to a fresh Eppendorf tube and 20 μL of ice‐cold extraction solvent (2:6:1 CHCl_3_:MeOH:H_2_O v/v/v with 2 μM CHAPS, CAPS, PIPES, and TRIS as internal standards) per mg of tissue was immediately added. The mixture was then briefly vortexed before sonication in an ice‐water bath for 10 min after which it was centrifuged (20,000 rcf, 4°C, 10 min). The supernatant was transferred to a mass spectrometry vial for subsequent liquid chromatography coupled to high‐resolution mass spectrometry (LC–MS) analysis.

### 4.8 Liquid chromatography coupled to high‐resolution mass spectrometry analysis

Samples were analyzed by hydrophilic interaction liquid chromatography coupled to high‐resolution mass spectrometry (LC–MS) according to a previously published method (Stoessel et al., 2016). In brief, chromatography was performed using a gradient elution of 20 mM ammonium carbonate (A) and acetonitrile (B) (linear gradient time—%B as follows: 0 min‐80%, 15 min‐50%, 18 min‐5%, 21 min‐5%, 24 min‐80%, and 32 min‐80%) at 25°C on a Dionex RSLC3000 UHPLC (Thermo Fisher Scientific) using a SeQuant ZIC‐p(HILIC) 150 × 4.6 mm column (Merck). The flow rate was maintained at 300 μL/min. Samples were kept between 4°C and 6°C in the autosampler and 10 μL injected for analysis. The mass spectrometry was performed on either a Q‐Exactive Orbitrap or a Q‐Exactive Plus MS (Thermo Fisher Scientific) operating in rapid switching positive (4 kV) and negative (−3.5 kV) mode electrospray ionization (capillary temperature 300°C; sheath gas 50; Aux gas 20; sweep gas 2; probe temperature 120°C). For each experiment, all samples were analyzed as a single batch, in random order and with pooled quality control, samples analyzed regularly throughout the batch to confirm reproducibility. ∼300 to 500 metabolite standards were analyzed immediately preceding the batch to determine accurate retention times to facilitate metabolite identification. Additional retention times for metabolites lacking authentic standards were predicted computationally as previously described (Creek et al., 2011).

### 4.9 Mitochondrial isolation and BN‐PAGE

Snap‐frozen killifish muscle was thawed in ice‐cold Solution A (20 mM HEPES‐KOH pH 7.6, 220 mM mannitol, 70 mM sucrose, 1 mM EDTA, 2 mg/mL fatty acid‐free bovine serum albumin (Sigma) with the addition of complete, EDTA‐free protease inhibitor cocktail tablet [Roche]) and homogenized with 20 strokes of a drill‐fitted Teflon pestle and glass homogenizer. The resulting homogenates were centrifuged at 800 g for 5 min at 4°C, and the supernatant collected and centrifuged at 10,000 g for 10 min at 4°C. The resulting pellet was collected and resuspended in ice‐cold Solution B (20 mM HEPES‐KOH pH 7.6, 220 mM mannitol, 70 mM sucrose, 1 mM EDTA and complete, and EDTA‐free Protease Inhibitor Cocktail Tablet [Roche, 11,873,580,001]). The samples were then clarified with centrifugation at 800 g for 5 min at 4°C, and the resulting supernatant collected and centrifuged at 10,000 g for 20 min at 4°C to obtain the mitochondrial pellet. The mitochondrial pellet was then resuspended in ice‐cold sucrose storage buffer (10 mM HEPES‐KOH pH 7.6, 0.5 M Sucrose) and mitochondrial protein concentration determined by the Pierce BCA Protein Assay Kit (Thermo Fisher Scientific) according to the manufacturer's instructions. Mitochondria were aliquoted and stored at −80°C until required.

BN‐PAGE was performed on isolated mitochondria as previously described McKenzie et al. (2007). Protein complexes separated by BN‐PAGE were transferred onto PVDF membrane (Merck) using a Power Blotter (Thermo Fisher Scientific) following the manufacturer's protocol. The membranes were then probed with: anti‐NDUFS3 (Molecular Probes, A21343, 1/1000), anti‐SDHB (a part of OXPHOS cocktail; Abcam, ab110413, 1/1000), anti‐ATP5A (a part of OXPHOS cocktail, Abcam, ab110413, 1/1000), or anti‐VDAC1 (in‐house antibody raised in rabbit, 1/500). Following this, the membrane was incubated in the appropriate HRP‐conjugated secondary antibody (Sigma‐Aldrich, A9044 or A0545, 1/5000). Immunoreactive complexes were detected using Clarify western ECL chemiluminescent substrate (BioRad; 1,705,061) and visualized using the BioRad ChemiDoc XRS+ imaging system. The blot images were quantified using Image Lab software (Bio‐Rad) and a one‐way ANOVA statistical test was used to test for significant changes in protein levels.

#### 4.9.1 Resveratrol drug treatments

Drug experiments were approved by the Monash Animal Ethics Committee (ERM33324) and were performed on 33‐week adults for 4 weeks, as per Valenzano et al., 2006 with a few modifications: 0.4 mg/μL resveratrol stock was prepared in 5% ethanol. Frozen Chironomus larvae were thawed, left to drip dry, and aliquoted into single portions of 500 μg, which is sufficient for five adult fish. A total quantity of 120 μL of the 0.4 mg/μL stock (or 5% ethanol for control animals) was added to each Chironomus aliquot, which was left at 4°C for 2 h to soak. A total quantity of 100 μL of 5% gelatin was added to the Chironomus/resveratrol aliquot, mixed, frozen, and stored at −20°C until use. For feeding, the frozen gelatin/Chironomus cube was thawed in water and fed to the fish, three times a day for 4 weeks. All uneaten food was removed after 20 min.

### 4.10 Quantification and statistical analysis

The number of independent biological replicates examined for each experiment, statistical tests used, and the associated *t*/F value, degrees of freedom (df), and exact *p*‐values obtained are detailed in Extended Data Table 4–10 in Data S1. For all experiments, the order in which data were captured was randomized, and the identity of the sample was blinded until after analyses were complete.

### 4.11 Quantification and visualization of muscle fiber size

Images were segmented using the interactive segmentation tool ilastik version 1.4.0b20 (Berg et al., 2019), following which the thresholding and participle analysis functions in Fiji (http://fiji.sc), were used to determine the sizes of individual muscle fibers. A total of 43,307 measurements were taken from two distinct anterior posterior positions, from 22 different animals. Each measurement was allocated to one of the three groups based on size: small (<1000 μm^2^), medium (between 1000 and 2600 μm^2^), and large (>2600 μm^2^) following which a generalized linear mixed model (with Bonferroni post hoc test) was used to identify any significant changes in probability of large fibers (vs medium fibers) and medium fibers (vs small fibers) across the four age groups. To visualize fiber size distribution, small, medium, and large fibers were colored in red, yellow, and green, respectively, using a custom‐written macro in Fiji. All images with the regions of interests annotated have been provided on Figshare.

### 4.12 Quantification of muscle stem cell number

A total quantity of 20–30 fibers from each size group, from each image were randomly selected using a custom‐written macro in Fiji. The number of Pax7+ muscle stem cells adjacent to selected fibers were then manually quantified using Fiji (http://fiji.sc). The number of Pax7+ muscle stem cells were normalized to the number of muscle cells and one‐way ANOVA with Tukey's multiple correction post hoc test was subsequently used to determine statistical significance.

### 4.13 Quantification of denervated endplates

The number of innervated endplates, identified by the presence of synaptic vesicle glycoprotein 2A and α‐bungarotoxin staining, and denervated endplates, identified by the absence of synaptic vesicle glycoprotein 2A staining, and the presence of α‐bungarotoxin staining, were manually quantified using Fiji. Chi‐squared test was used to identify statistically significant changes in the proportion of innervated/denervated endplates at each of the four times.

### 4.14 Quantification of lipid droplet distribution based on Bodipy (493/503) staining

Small, medium, and large muscle fibers were classified as above, following which 20–30 muscle cells from each group was randomly selected (using the randomizer macro described above) in each fish, and allocated to one of four groups: no apparent staining, predominantly subsarcolemmal (SS) staining, predominantly intermyofibrillar (IM) or equal distribution of SS and IM localization. Two‐way ANOVA with Tukey's multiple correction post hoc test was subsequently used to identify any statistically significant changes in each of the four lipid distribution patterns across the three time points.

### 4.15 Data analyses for metabolomics experiment

Untargeted metabolomics data were analyzed using the IDEOM (version 20) workflow with default parameters (Creek et al., 2012). In brief, this involved peak picking with XCMS (Tautenhahn et al., 2008), peak alignment and filtering with mzMatch (Scheltema et al., 2011) and further filtering, and metabolite identification with IDEOM. The dataset was deduplicated by selecting the duplicate metabolite with the highest confidence value, or where the confidence value is the same, the metabolite with maximum intensity was selected and others were filtered out. For comparative analyses, the metabolomics raw dataset was transformed to a log‐10 scale, and subsequently principle component analysis (PCA) and hierarchical clustering were performed using the web‐based tool MetaboAnalyst 3.0 (Chong et al., 2019). For trend analyses, the raw dataset was transformed to a log‐2 scale following which the variations of abundance of metabolites across three time points were assessed based on four trend groups including upregulation, downregulation, bell‐shaped, and U‐shaped which altogether consisted of 17 different trend types. For this purpose, a one‐way analysis of variance (ANOVA) was applied on the metabolomics data followed by a Tukey's HSD (honestly significant difference) test for multiple pairwise comparisons, and each metabolite was assigned a trend type based on its mean abundance in different time points. For all of these analyses, *p*‐value <0.05 was considered as statistically significant.

The co‐abundance of metabolites was assessed via calculation of correlation of the abundance of metabolites across all samples of all time points using the Pearson algorithm. Next, all of the paired metabolites with Pearson correlation coefficient (*r*) >0.7 and adjusted *p*‐value <0.01 were selected as significant co‐abundant metabolites. Subsequently, an association network was reconstructed based on the co‐abundance of metabolites using the Cytoscape software v.3.7.1 (Shannon et al., 2003). The topology of network was analyzed using the Cytoscape software and Markov Clustering algorithm (MCL) was used for the identification of functional modules of metabolites. Also, the integrated value of influence (IVI) of each metabolite was calculated based on the IVI method (Salavaty et al., 2020), using the “influential” R package (https://cran.r‐project.org/package=influential). IVI scores of all metabolites is provided in the Figshare repository. The putative identity of the top 20 most influential metabolites were confirmed by manual curation of the LC–MS data, which resulted in removal of five of these metabolites that were identified as mass spectrometry artifacts. As such only 15 metabolites have been presented. Furthermore, a network was reconstructed based on the metabolites belonging to the trend types of bell‐shaped group and the IVI of each node was calculated similarly.

## AUTHOR CONTRIBUTION

Conceptualization, A.A.R and P.D.C; Methodology: A.A.R, C.K.B, Y.L, M.J.E, D.J.C, and R.B.S; Software: A.S, C.K.B, Y.L, D.C, and M.R; Formal analyses, A.AR, A.S, C.K.B, Y.L, M.J.E, and D.J.C; Investigation: A.A.R, Y.L, C.S, L.H, C.K.B, and M.J.E; Writing—Original Draft, A.A.R; Writing—review and editing, A.A.R and P.D.C; Resources: C.E; Visualization: A.A.R; Supervision: A.A.R, R.B.S, M.R, M.T.R, C.E, and P.D.C.; Project Administration, A.A.R and P.D.C; Funding Acquisition: A.A.R and P.D.C.

## CONFLICT OF INTEREST STATEMENT

The authors declare no competing interests.

### OPEN RESEARCH BADGES

This article has earned an Open Data badge for making publicly available the digitally‐shareable data necessary to reproduce the reported results. The data is available at [https://figshare.com/s/868d6bdef790f79dbd3d].

## Supporting information


Data S1

## Data Availability

Raw data, including the metabolomics datasets, and files for statistical analysis are available at Figshare and can be accessed using the following link: https://figshare.com/s/868d6bdef790f79dbd3d.
