# Supplementary material for: The African killifish: A short‐lived vertebrate model to study the biology of sarcopenia and longevity
Source: Aging Cell. 2023 May 14;23(1):e13862. doi: 10.1111/acel.13862 (PMC10776123; doi:10.1111/acel.13862)
Supplement: Supplementary file 1 — Data S1 [file ACEL-23-e13862-s001.pdf]

Extended Data

The African killifish: a short-lived vertebrate model to study the biology of sarcopenia and longevity

Avnika A. Ruparelia[1,2,3*], Abbas Salavaty[1,4], Christopher K. Barlow[5,6], Yansong Lu[1], Carmen Sonntag[1], Lucy Hersey[1], Matthew J. Eramo[7], Johannes Krug[8], Hanna Reuter[8], Ralf B. Schittenhelm[5,6], Mirana Ramialison[1,4], Andrew Cox[9,10], Michael T. Ryan[7], Darren J. Creek[6,11], Christoph Englert[8,12], Peter D. Currie[1*].

[1] Australian Regenerative Medicine Institute, Monash University, Wellington Road, Clayton, VIC 3800, Australia.
[2] Department of Anatomy and Physiology, School of Biomedical Sciences, Faculty of Medicine Dentistry and Health Sciences, University of Melbourne, Melbourne, Victoria, 3010, Australia.
[3] Centre for Muscle Research, Department of Anatomy and Physiology, University of Melbourne, Melbourne, Victoria, 3010, Australia
[4] Systems Biology Institute Australia, Monash University, Clayton, VIC 3800, Australia.
[5] Department of Biochemistry and Molecular Biology, Monash University, Clayton, Victoria 3800, Australia.
[6] Monash Proteomics and Metabolomics Facility, Monash Biomedicine Discovery Institute, Monash University, Clayton, Victoria 3800, Australia.
[7] Department of Biochemistry and Molecular Biology, Monash Biomedicine Discovery Institute, Monash University, Clayton, Victoria 3800, Australia.
[8] Leibniz Institute on Aging – Fritz Lipmann Institute (FLI), 07745 Jena, Germany.
[9] Peter MacCallum Cancer Centre, Melbourne, Victoria, Australia.
[10] Department of Biochemistry and Pharmacology, The University of Melbourne, Melbourne, VIC, Australia.
[11] Drug Delivery, Disposition and Dynamics, Monash Institute of Pharmaceutical Sciences, Monash University, Parkville, Victoria 3052, Australia.
[12] Institute of Biochemistry and Biophysics, Friedrich-Schiller-University Jena, 07745 Jena, Germany

*Authors for Correspondence:
Avnika A. Ruparelia - avnika.ruparelia@unimelb.edu.au
Peter D. Currie - peter.currie@monash.edu

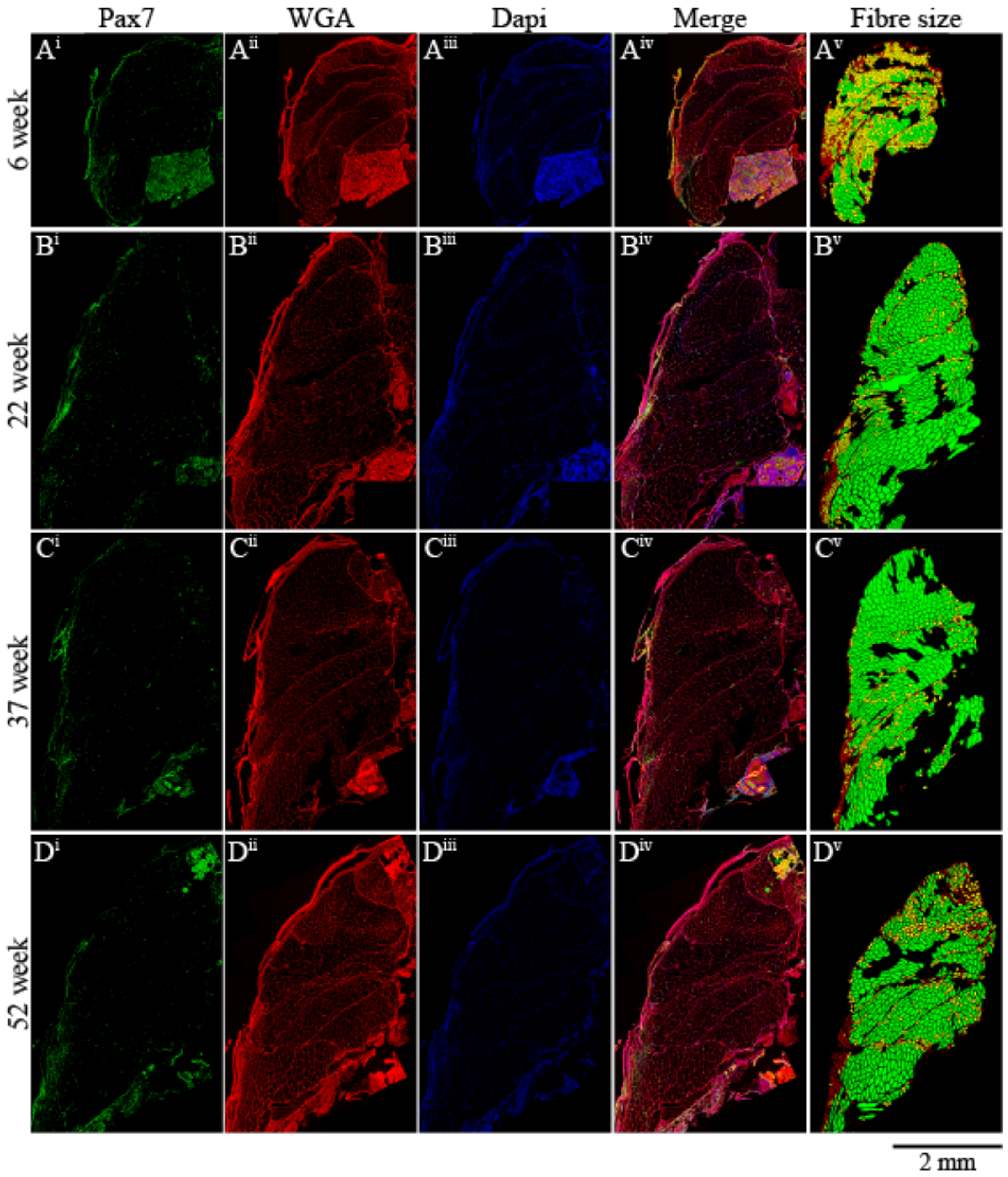

Extended Data Fig. 1: Localization of muscle stem cells in killifish. Representative images of Pax7, conjugated wheat gern agglutinin (WGA) and Dapi labelling on muscle from 6-week (a), 22-week (b), 37-week (c) and 52-week (d) old male killifish. Heat map displaying fibre size distribution is also included.

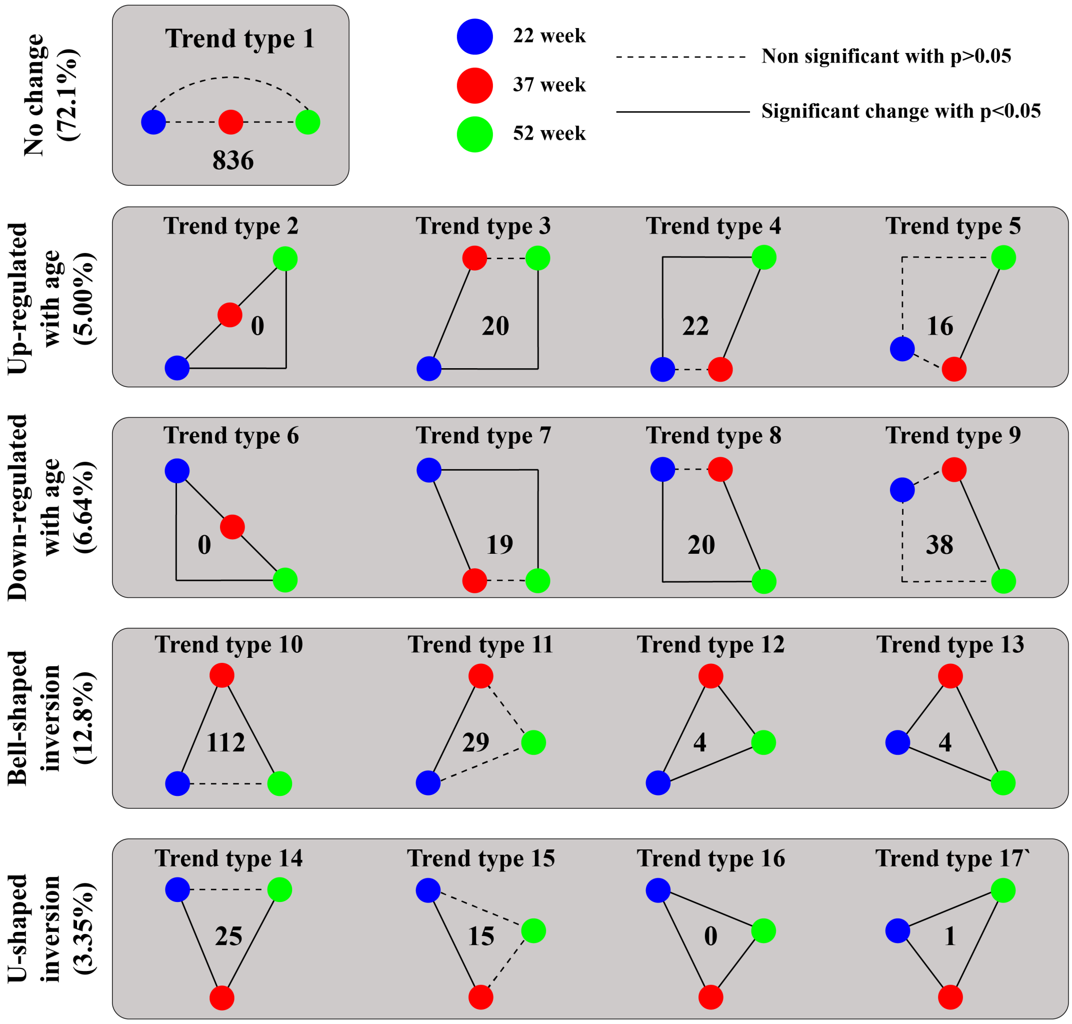

Extended Data Fig. 2: Schematic of trends presented by metabolites. Metabolites statistically fall in one of five broad groups covering 17 possible trend types. Trend type 1 covers metabolites that are unaltered with age; metabolites in group two are upregulated with age and within this group they follow one of four patterns (trend 2-5). Similarly, metabolites in group three are downregulated with age covering trends 6-9. The fourth group of metabolites, consisting of trends 10-13, show a bell-shaped inversion where by the abundance of the metabolite at 37-week is higher than 22-week-week and 52-week. The final set of metabolites display a U-shaped inversion in that the abundance of the metabolite at 37-week is lower than 22-week-week and 52-week, covering trends 14-17. 22-week-week samples displayed in blue, 37-week cohort in red, and 52-week fish shown in green. Dashed lines represent non-significant changes with $p>0.05$, and solid lines reflect significant alterations with $p<0.05$.

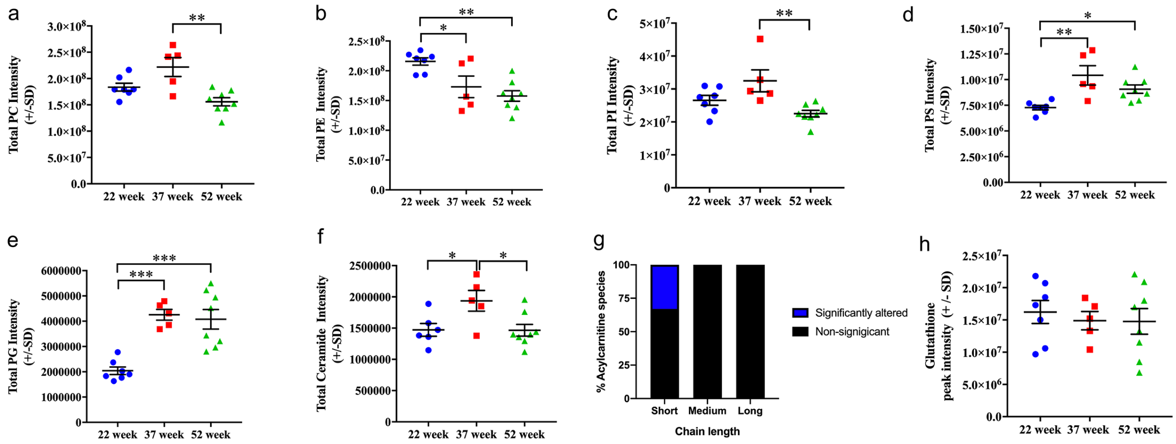

Extended Data Fig. 3: Metabolic alterations in aged and late-life killifish. Total intensity of phosphatidylcholine (PC; a), phosphatidylethanolamine (PE; b), phosphatidylinositol (PI; c), phosphatidylserine (PS; d), phosphatidylglycerol (PG; e) and ceramides (f) in 22-week week early-life, 37-week aged and 52-week late-life fish. (g) Graph depicting the distribution of significantly affected short (C2-C6), medium (C7-C12) and long chain (C13-C22) acylcarnitine species. (h) Peak intensity of antioxidant Glutathione in 22-week week early-life, 37-week aged and 52-week late-life fish. Error bars represent +/- SD. * p<0.05; ** p<0.01; *** p<0.001 calculated using one way ANOVA with Tukey's multiple correction post hoc test.

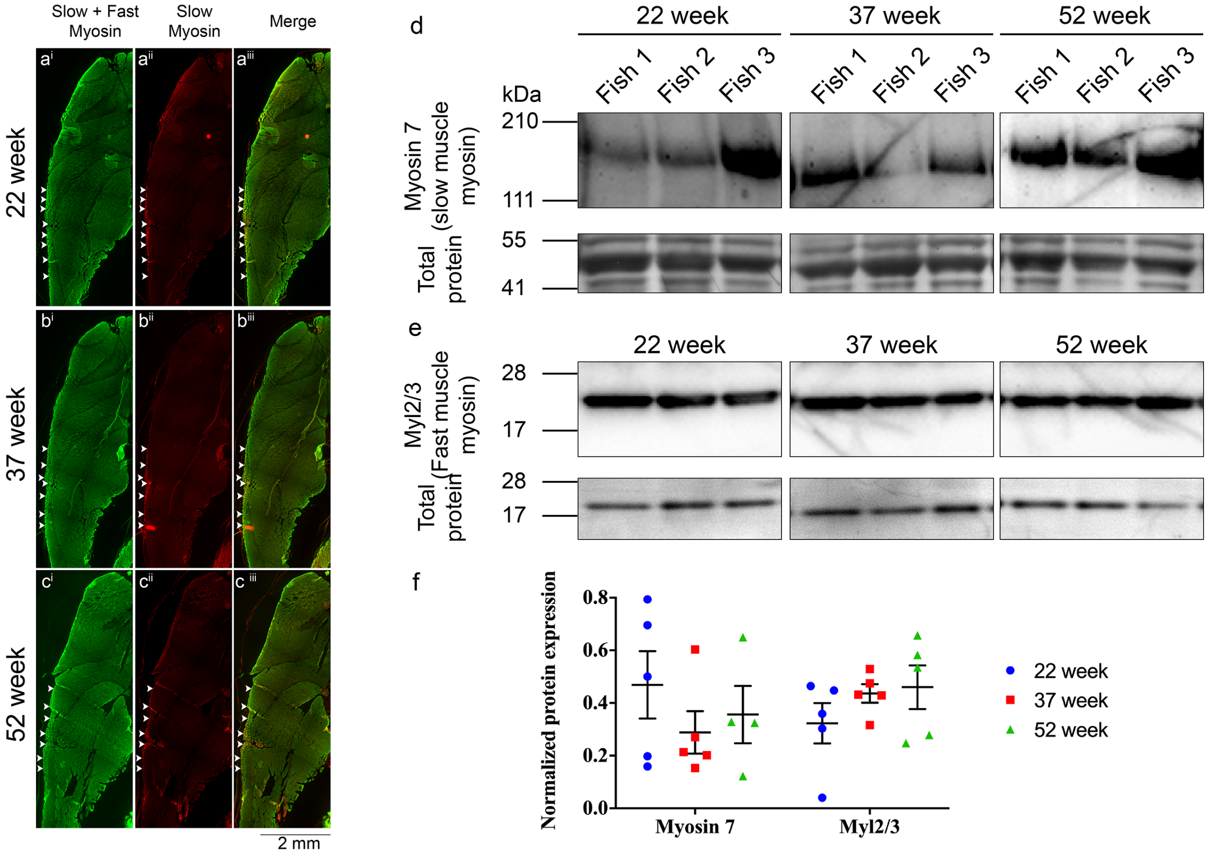

Extended Data Fig. 4: Aged killifish show no evidence of fibre type switching. Representative tile-scan images of slow and fast muscle Myosin labelling, and slow muscle Myosin labelling in 22-week week (a), 37-week (b) and 52-week (c) old male killifish. Arrowheads indicate slow muscle cells. Western blot for Myosin 7 (slow muscle myosin, d) and respective total protein direct blue stain, and Myosin light chain 2/3 (Myl2/3; fast muscle myosin; e) and respective total protein direct blue stain, on muscle lysates from three 22-week week, 37-week and 52-week old male killifish. To accurately reflect the variation observed in LC3 and p62 levels, three represented samples from each stage are displayed. (f) Quantification Myosin 7 and (C) Myosin light chain 2/3 (Myl2/3) protein levels. Error bars represent +/- SD.

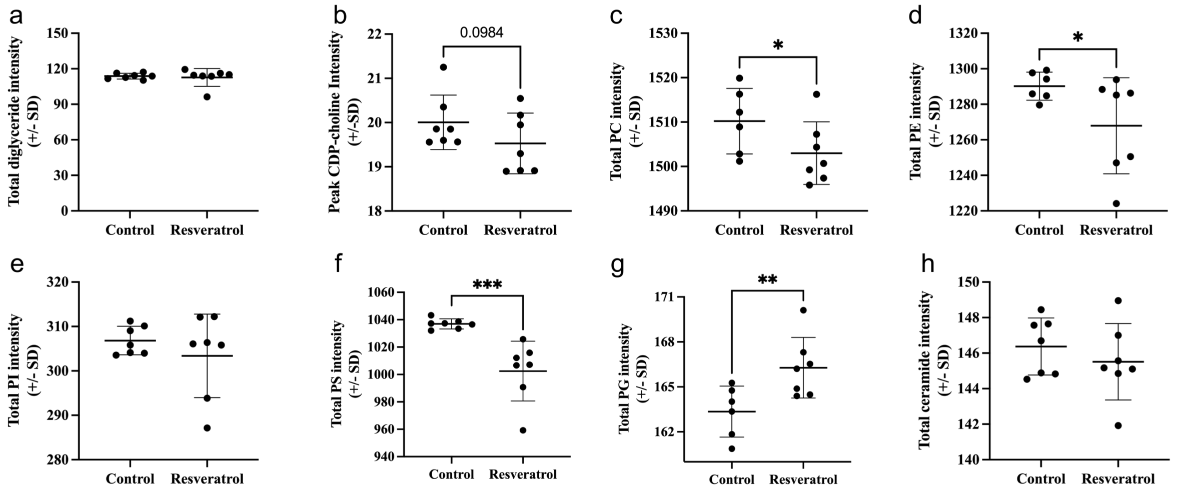

Extended Data Fig. 5 Resveratrol treatment and subsequent *sirt1* upregulation results in a late-life like lipid profile. Total intensity of diglycerides (DG; a). CDP-choline (b), phosphatidylcholine (PC; c), phosphatidylethanolamine (PE; d), phosphatidylinositol (PI; e), phosphatidylserine (PS; f), phosphatidylglycerol (PG; g) and ceramides (h) in 37 week control and resveratrol treated animals. Error bars represent +/- SD. * $p<0.05$; ** $p<0.01$; *** $p<0.001$ calculated using students T-test.

Extended Data Table 1: List of the top 15 most influential putative metabolites determined using our "Integrated Value of Significance" (IVI) algorithm. The IVI score reflects the authoritative position the metabolite holds within the network, with larger scores reflecting a more influential position. The trend column is based on the ANOVA analyses and highlights how the metabolite is altered with across the lifespan of the fish.

Putative metabolite	IVI	Trend	Pathway
PC(38:8)	100.0	10: Bell-shaped inversion	Lipid
5,6-dihydrouracil	61.9	10: Bell-shaped inversion	Nucleotide
Methylmalonylcarnitine/succinylcarnitine	61.1	10: Bell-shaped inversion	Lipid
Hydroxy-octadecenoylcarnitine	60.4	1: No change	Lipid
PC(40:10)	59.3	10: Bell-shaped inversion	Lipid
Carbamyl-arginine	56.8	10: Bell-shaped inversion	Microbial
CL(72:9)	49.7	10: Bell-shaped inversion	Lipid
PC(34:4)	49.5	10: Bell-shaped inversion	Lipid
NADP+	48.7	10: Bell-shaped inversion	Energy
PI(36:3)	48.2	10: Bell-shaped inversion	Lipid
PC(34:3)	46.4	10: Bell-shaped inversion	Lipid
PC(40:8)	44.6	10: Bell-shaped inversion	Lipid
Hexanoylcarnitine	38.9	1: No change	Lipid
Methylgluconamide	38.6	1: No change	Unclassified
PC(38:9)	37.4	10: Bell-shaped inversion	Lipid

Extended Data Table 2: Mean peak intensity (+/- standard deviation) of metabolites involved in carbohydrate metabolism. With the exception of succinate (highlighted), which was significantly reduced in 52 week late-life fish, all other metabolites fell into trend 1 highlighting that they are not significantly altered at any of the time-points.

Pathway	Metabolotie	Trend	22 week		37 week		52 week	
			Mean	+/- SD	Mean	+/- SD	Mean	+/- SD
Glycolysis	Glucose	1: No change	3501607.000	2665094.000	3116773.000	2270295.000	6029469	5566327
	Glucose 6-phosphate	1: No change	63914286	28502773	43700000	14334399	57600000	30637279
	Fructose 6-phosphate	1: No change	18753939	9176297	14115996	4043308	22013061	11574952
	Fructose 1,6-bisphosphate	1: No change	142328	62528	140891	74111	199263	61080
	Phospho-D-glycerate	1: No change	1580977	489305	3123168	1847690	1863882	815494
	Propylphosphoenolpyruvate	1: No change	47585	19350	95315	59670	67594	32760
	Pyruvate	1: No change	504456	253330	483623	250765	579941	449613
	Lactate	1: No change	64057143	19928193	44200000	24817232	59587500	17151130
Fructose and Mannose metabolism	Mannose	1: No change	294058	88843	255436	236055	496520	243083
	Fructose	1: No change	3071290	1623805	2140660	1580270	4642757	2567573
Pentose Phosphate pathway	6-Phosphogluconate	1: No change	50639	34157	57661	28267	45963	31688
	Ribose 5-phosphate	1: No change	398503	294636	591129	652954	591240	227566
	Ribulose	1: No change	75446	49911	125438	95907	71352	44963
	Ribose	1: No change	4711533	3558410	1320718	185695	5400909	3984004
Tricarboxylic acid cycle	Citrate	1: No change	11212403	1908656	9718635	4071569	11484618	1646878
	cis Acionate	1: No change	272679	82106	229027	74827	295925	63363
	alpha Ketoglutaric acid	1: No change	1786559	1315625	1483244	1167117	461733	297496
	Succinate	7: Down regulated	8803509	3143465	4638867	3363322	2857766	1086343
	Malate	1: No change	62757143	14689549	51380000	12528248	40175000	15475580

Extended Data Table 3: Sequences of all primers used in the study

PRIMER NAME	PRIMER SEQUENCE (5'-3')
actb_F	ctcttccagccttccttcct
actb_R	cagggctgtgatctccttct
GAPDH_F	ggagaaaccagccaagtacg
GAPDH_R	tgctgtagccgaactcattg
Atrogin1_F	gcctgctgtggaagaaactc
Atrogin1_R	ctctctgggttgttggctgt
Murf1_F	ccaaaggcctcatcacaaag
Murf1_R	catcatcaccagcaccaaag
chrna1_F	cctccacttccagtgctgtt
chrna1_R	ttgtttcgatgaaaaccttgc
chrnb1_F	ggcagaaaaaggttgtcacc
chrnb1_R	gatggcttcaatggctgatt
chrng_F	gcctctccagagctgaaaca
chrng_R	agggaggctggttgaaatg
chrnd_F	cagctgtagatggagcgaact
chrnd_R	agtcgtgtgggtctcctttg
myog_F	gacagacggggttgcactac
myog_R	tcagctccagtgatgctgtc
myf5_F	cagacagcctggagtttggt
myf5_R	cctcaaaggcatggttgact
Myod_F	ccatgttcgagcagaagtga
Myod_R	gatggtgggggtgttatgag
CD36_F	ggcaaaggtgtacaggcagt
CD36_R	aatcagaaaggaggcggttt
Cpt1b_F	ggcgtggaagaagaaagcta
Cpt1b_F	ggcaggttgtccttcatcag
Cpt2_F	atctgggagtcgatttcgtg
Cpt2_R	tcaagtttcggaatgggaag
GPX1_F	tactctgctcaggggcttgt
GPX1_R	cccttcccattcacattcac
SOD1_F	cttgctgatggtgaacatgg
SOD1_R	cagtcacattccccaggtct
SOD2_F	aggctcttgcaaagggagat
SOD2_R	ttgatggcctccataagctc

Extended Data Table 4: Number of samples examined for each experiment

Figure	Description	Number of samples			
		6 week	22 week	37 week	52 week
1b	Body weight	25	27	25	30
1c	Body length	25	27	25	30
1f-h	Fibre cross-sectional area	11016 fibres (5 fish)	9197 fibres (6 fish)	13808 fibres (6 fish)	9286 fibres (5 fish)
2a	qRT-PCR atrogin1 and murf1	6	6	5	6
2b-2c	LC3 and p62 western blot	5	6	5	6
2e-f	Stem cell number	8	9	9	9
2g-h	Dennervation	6	5	6	6
2i-j	qRT-PCR	6	6	5	6
4	Metabolomics	-	7	5	8
5a-d	Lipid metabolites	-	7	5	8
5e-f	qRT-PCR	-	6	5	6
5h-i	VDAC1 western blot	-	6	4	5
5j-m	BN-PAGE	-	6	5	6
5n-o	ROS metabolites	-	7	5	8
5p-r	qRT-PCR	-	6	5	6
6	Lipid stains	-	6	6	6
7	Control vs resveratrol	-	-	7 Control<br>7 Resveratrol	
Extended data 2	Metabolomics trends	-	7	5	8
Extended data 3	Lipid and ROS metabolites	-	7	5	8
Extended data 4a	Fibre type	-	3	3	3
Extended data 4d and 4f	Western blot Myosin 7	-	5	5	4
Extended data 4e-f	Western blot Myl2/3	-	5	5	5
Extended data data 5	Control vs resveratrol	-	-	7 Control<br>7 Resveratrol	

Extended Data Table 5: Statistical tests for experiments presented in Fig. 1

Figure	Description	Test used	t/F value	degrees of freedom (df)	Multiple comparison	p values (adjusted)
1b	Body weight	One way ANOVA with Tukey's multiple correction post hoc test	63.79	103	6 week vs. 22 week	<0.0001
					6 week vs. 37 week	<0.0001
					6 week vs. 52 week	<0.0001
					22 week vs. 37 week	0.2202
					22 week vs. 52 week	0.007
					37 week vs. 52 week	0.5925
1c	Body weight	One way ANOVA with Tukey's multiple correction post hoc test	79.38	103	6 week vs. 22 week	<0.0001
					6 week vs. 37 week	<0.0001
					6 week vs. 52 week	<0.0001
					22 week vs. 37 week	0.9061
					22 week vs. 52 week	<0.0001
					37 week vs. 52 week	0.0006
1f	Fibre size: Large vs Medium	Generalised linear mixed model with Bonferroni multiple correction post hoc test	48.826	30454	6 week vs. 22 week	<0.0001
					6 week vs. 37 week	0.164
					6 week vs. 52 week	0.016
					22 week vs. 37 week	0.066
					22 week vs. 52 week	0.491
					37 week vs. 52 week	0.491
1g	Fibre size: Medium vs Small	Generalised linear mixed model with Bonferroni multiple correction post hoc test	6.457	28295	6 week vs. 22 week	<0.0001
					6 week vs. 37 week	<0.0001
					6 week vs. 52 week	<0.0001
					22 week vs. 37 week	0.182
					22 week vs. 52 week	0.008
					37 week vs. 52 week	0.182
1h	Fibre number	One way ANOVA with Tukey's multiple correction post hoc test	10.87	16	6 week vs. 22 week	0.0005
					6 week vs. 37 week	0.0016
					6 week vs. 52 week	0.0093
					22 week vs. 37 week	0.6896
					22 week vs. 52 week	0.36
					37 week vs. 52 week	0.8988

Extended Data Table 6: Statistical tests for experiments presented in Fig. 2

Figure	Description	Test used	t/F value	degrees of freedom (df)	Multiple comparison	p values (adjusted)
2a	qRT-PCR atrogin1 and murf1	Two way ANOVA with Tukey's multiple correction post hoc test	-	38	atrogin1: 6 week vs. 22 week	<0.0001
					atrogin1: 6 week vs. 37 week	<0.0001
					atrogin1: 6 week vs. 52 week	<0.0001
					atrogin1: 22 week vs. 37 week	0.2305
					atrogin1: 22 week vs. 52 week	0.2492
					atrogin1: 37 week vs. 52 week	0.0033
					murf1: 6 week vs. 22 week	0.0031
					murf1: 6 week vs. 37 week	0.0028
					murf1: 6 week vs. 52 week	0.001
					murf1: 22 week vs. 37 week	0.9968
					murf1: 22 week vs. 52 week	0.9776
					murf1: 37 week vs. 52 week	0.998
2c	LC3 western blot	One way ANOVA with Tukey's multiple correction post hoc test	4.125	18	6 week vs. 22 week	0.8661
					6 week vs. 37 week	0.9996
					6 week vs. 52 week	0.1097
					22 week vs. 37 week	0.818
					22 week vs. 52 week	0.0173
					37 week vs. 52 week	0.132
2d	p62 western blot	One way ANOVA with Tukey's multiple correction post hoc test	10.34	18	6 week vs. 22 week	0.7812
					6 week vs. 37 week	0.1816
					6 week vs. 52 week	0.0049
					22 week vs. 37 week	0.0249
					22 week vs. 52 week	0.0004
					37 week vs. 52 week	0.3469
2i	Stem cell number (total)	One way ANOVA with Tukey's multiple correction post hoc test	2.787	31	6 week vs. 22 week	0.6052
					6 week vs. 37 week	0.9709
					6 week vs. 52 week	0.4344
					22 week vs. 37 week	0.3233
					22 week vs. 52 week	0.0363
					37 week vs. 52 week	0.6807
2j	Stem cell number (small)	One way ANOVA with Tukey's multiple correction post hoc test	3.857	29	6 week vs. 22 week	0.4628
					6 week vs. 37 week	0.9791
					6 week vs. 52 week	0.2524
					22 week vs. 37 week	0.2386
					22 week vs. 52 week	0.0108
					37 week vs. 52 week	0.4121
2k	Stem cell number (medium)	One way ANOVA with Tukey's multiple correction post hoc test	0.9885	30	6 week vs. 22 week	0.8753
					6 week vs. 37 week	0.8566
					6 week vs. 52 week	0.3348
					22 week vs. 37 week	>0.9999
					22 week vs. 52 week	0.7838
					37 week vs. 52 week	0.7796
2l	Stem cell number (large)	One way ANOVA with Tukey's multiple correction post hoc test	2.816	31	6 week vs. 22 week	0.7085
					6 week vs. 37 week	0.9932
					6 week vs. 52 week	0.329
					22 week vs. 37 week	0.5201
					22 week vs. 52 week	0.0341
					37 week vs. 52 week	0.4492
2n	Dennervation	Chi squared test	133. 9 (Chi-square)	3	N/A	<0.0001

2o	qRT-PCR	Two way ANOVA with Tukey's multiple correction post hoc test	-	76	chrna1: 6 week vs. 22 week	0.8857
					chrna1: 6 week vs. 37 week	0.9995
					chrna1: 6 week vs. 52 week	0.991
					chrna1: 22 week vs. 37 week	0.9367
					chrna1: 22 week vs. 52 week	0.7367
					chrna1: 37 week vs. 52 week	0.9795
					chrnb1: 6 week vs. 22 week	0.9939
					chrnb1: 6 week vs. 37 week	0.7431
					chrnb1: 6 week vs. 52 week	0.9152
					chrnb1: 22 week vs. 37 week	0.8695
					chrnb1: 22 week vs. 52 week	0.9793
					chrnb1: 37 week vs. 52 week	0.9795
					chrng: 6 week vs. 22 week	0.6247
					chrng: 6 week vs. 37 week	0.7441
					chrng: 6 week vs. 52 week	0.9746
					chrng: 22 week vs. 37 week	0.999
					chrng: 22 week vs. 52 week	0.8613
					chrng: 37 week vs. 52 week	0.9294
					chrnd: 6 week vs. 22 week	0.9248
					chrnd: 6 week vs. 37 week	0.1057
					chrnd: 6 week vs. 52 week	0.0046
					chrnd: 22 week vs. 37 week	0.324
					chrnd: 22 week vs. 52 week	0.0281
					chrnd: 37 week vs. 52 week	0.7469
2p	qRT-PCR	Two way ANOVA with Tukey's multiple correction post hoc test	-	57	myog: 6 week vs. 22 week	0.2873
					myog: 6 week vs. 37 week	0.0008
					myog: 6 week vs. 52 week	0.9996
					myog: 22 week vs. 37 week	<0.0001
					myog: 22 week vs. 52 week	0.3398
					myog: 37 week vs. 52 week	0.0006
					myf5: 6 week vs. 22 week	0.9409
					myf5: 6 week vs. 37 week	0.0457
					myf5: 6 week vs. 52 week	0.7216
					myf5: 22 week vs. 37 week	0.1528
					myf5: 22 week vs. 52 week	0.377
					myf5: 37 week vs. 52 week	0.0028
					myoD: 6 week vs. 22 week	0.3089
					myoD: 6 week vs. 37 week	0.0006
					myoD: 6 week vs. 52 week	0.9824
					myoD: 22 week vs. 37 week	0.0688
					myoD: 22 week vs. 52 week	0.5187
					myoD: 37 week vs. 52 week	0.0018

Extended Data Table 7: Statistical tests for experiments in presented Fig. 5

Figure	Description	Test used	t/F value	degrees of freedom (df)	Multiple comparison	p values (adjusted)
5b	Triglyceride levels	One way ANOVA with Tukey's multiple correction post hoc test	9.898	17	22 week vs. 37 week	0.0042
					22 week vs. 52 week	0.9474
					37 week vs. 52 week	0.0018
5c	Diglyceride levels	One way ANOVA with Tukey's multiple correction post hoc test	12.44	18	22 week vs. 37 week	0.0003
					22 week vs. 52 week	0.2089
					37 week vs. 52 week	0.0082
5d	CDP choline levels	One way ANOVA with Tukey's multiple correction post hoc test	13.42	17	22 week vs. 37 week	0.0005
					22 week vs. 52 week	0.8653
					37 week vs. 52 week	0.001
5e	qRT-PCR CD36	One way ANOVA with Tukey's multiple correction post hoc test	6.072	14	22 week vs. 37 week	0.878
					22 week vs. 52 week	0.0355
					37 week vs. 52 week	0.018
5f	qRT-PCR cpt1b	One way ANOVA with Tukey's multiple correction post hoc test	5.453	14	22 week vs. 37 week	0.9153
					22 week vs. 52 week	0.0437
					37 week vs. 52 week	0.0259
5f	qRT-PCR cpt2	One way ANOVA with Tukey's multiple correction post hoc test	3.121	14	22 week vs. 37 week	0.9665
					22 week vs. 52 week	0.1331
					37 week vs. 52 week	0.1024
5g	Acyl carnatines	One way ANOVA with Tukey's multiple correction post hoc test	8.633	17	22 week vs. 37 week	0.0832
					22 week vs. 52 week	0.0019
					37 week vs. 52 week	0.3661
5i	VDAC1	One way ANOVA with Tukey's multiple correction post hoc test	7.092	12	22 week vs. 37 week	0.9873
					22 week vs. 52 week	0.0151
					37 week vs. 52 week	0.0208
5k	NDUFS3	One way ANOVA with Tukey's multiple correction post hoc test	31.38	14	22 week vs. 37 week	<0.0001
					22 week vs. 52 week	<0.0001
					37 week vs. 52 week	0.4159
5l	SDHB	One way ANOVA with Tukey's multiple correction post hoc test	5.71	14	22 week vs. 37 week	0.191
					22 week vs. 52 week	0.2748
					37 week vs. 52 week	0.0117
5m	ATP5A	One way ANOVA with Tukey's multiple correction post hoc test	2.1	15	22 week vs. 37 week	0.1398
					22 week vs. 52 week	0.7354
					37 week vs. 52 week	0.4314

5n	Glutathione disulphide	One way ANOVA with Tukey's multiple correction post hoc test	6.824	17	22 week vs. 37 week	0.0169
					22 week vs. 52 week	0.9499
					37 week vs. 52 week	0.0079
5o	Cysteinyl glutathione	One way ANOVA with Tukey's multiple correction post hoc test	11.46	17	22 week vs. 37 week	0.0026
					22 week vs. 52 week	0.8908
					37 week vs. 52 week	0.0009
5p	qRT PCR GPX1	One way ANOVA with Tukey's multiple correction post hoc test	7.326	14	22 week vs. 37 week	0.0121
					22 week vs. 52 week	>0.9999
					37 week vs. 52 week	0.012
5q	qRT PCR SOD1	One way ANOVA with Tukey's multiple correction post hoc test	8.778	14	22 week vs. 37 week	0.0116
					22 week vs. 52 week	0.8435
					37 week vs. 52 week	0.0041
5q	qRT PCR SOD2	One way ANOVA with Tukey's multiple correction post hoc test	8.748	14	22 week vs. 37 week	0.0357
					22 week vs. 52 week	0.3675
					37 week vs. 52 week	0.0027
5r	qRT PCR SIRT1	One way ANOVA with Tukey's multiple correction post hoc test	8.748	14	22 week vs. 37 week	0.0357
					22 week vs. 52 week	0.3675
					37 week vs. 52 week	0.0027
5r	qRT PCR PGC1	One way ANOVA with Tukey's multiple correction post hoc test	8.68	14	22 week vs. 37 week	0.9981
					22 week vs. 52 week	0.0064
					37 week vs. 52 week	0.01

Extended Data Table 8: Statistical tests for experiments in presented Fig. 6

Figure	Description	Test used	t/F value	degrees of freedom (df)	Multiple comparison	p values (adjusted)
6d	Lipid distribution (total)	Two way ANOVA with Tukey's multiple correction post hoc test	-	36	No apparent staining	
					22 week vs. 37 week	<0.0001
					22 week vs. 52 week	0.001
					37 week vs. 52 week	<0.0001
					Predominantly IM	
					22 week vs. 37 week	0.485
					22 week vs. 52 week	0.2656
					37 week vs. 52 week	0.025
					Predominantly SS	
					22 week vs. 37 week	0.7898
					22 week vs. 52 week	0.7221
					37 week vs. 52 week	0.3369
					Equal IM and SS	
					22 week vs. 37 week	<0.0001
					22 week vs. 52 week	<0.0001
					37 week vs. 52 week	0.0001
6e	Lipid distribution (small)	Two way ANOVA with Tukey's multiple correction post hoc test	-	36	No apparent staining	
					22 week vs. 37 week	<0.0001
					22 week vs. 52 week	0.002
					37 week vs. 52 week	0.0001
					Predominantly IM	
					22 week vs. 37 week	>0.9999
					22 week vs. 52 week	0.9001
					37 week vs. 52 week	0.9001
					Predominantly SS	
					22 week vs. 37 week	0.5361
					22 week vs. 52 week	0.8228
					37 week vs. 52 week	0.2302
					Equal IM and SS	
					22 week vs. 37 week	<0.0001
					22 week vs. 52 week	0.0023
					37 week vs. 52 week	<0.0001
6f	Lipid distribution (medium)	Two way ANOVA with Tukey's multiple correction post hoc test	-	36	No apparent staining	
					22 week vs. 37 week	<0.0001
					22 week vs. 52 week	0.014
					37 week vs. 52 week	0.0075
					Predominantly IM	
					22 week vs. 37 week	0.7786
					22 week vs. 52 week	0.1076
					37 week vs. 52 week	0.024
					Predominantly SS	
					22 week vs. 37 week	0.4201
					22 week vs. 52 week	0.9605
					37 week vs. 52 week	0.2838
					Equal IM and SS	
					22 week vs. 37 week	<0.0001
					22 week vs. 52 week	<0.0001
					37 week vs. 52 week	0.1263

6g	Lipid distribution (large)	Two way ANOVA with Tukey's multiple correction post hoc test	-	36	No apparent staining	
					22 week vs. 37 week	<0.0001
					22 week vs. 52 week	0.087
					37 week vs. 52 week	0.0012
					Predominantly IM	
					22 week vs. 37 week	0.5374
					22 week vs. 52 week	0.7393
					37 week vs. 52 week	0.1789
					Predominantly SS	
					22 week vs. 37 week	0.9872
					22 week vs. 52 week	0.9565
					37 week vs. 52 week	0.9906
					Equal IM and SS	
					22 week vs. 37 week	<0.0001
					22 week vs. 52 week	0.0298
					37 week vs. 52 week	0.087

Extended Data Table 9: Statistical tests for experiments in presented Fig. 7

Figure	Description	Test used	t/F value	degrees of freedom (df)	Multiple comparison	p values (adjusted)
7a	Body length	Unpaired t test (two-tailed)	0.1255	11	Control vs resveratrol	0.9024
7b	Body weight	Unpaired t test (two-tailed)	2.303	11	Control vs resveratrol	0.0418
7c	sirt1 mRNA	Unpaired t test (one-tailed)	2.183	11	Control vs resveratrol	0.0258
7e	TG levels	Unpaired t test (one-tailed)	2.133	12	Control vs resveratrol	0.0271
7h	Lipid distributio	Unpaired t test with Holm-Šídák method multiple comparison	-	10	No apparent staining	
					Control vs resveratrol	0.015118
					Predominantly IM	
					Control vs resveratrol	0.306171
					Predominantly SS	
					Control vs resveratrol	0.798854
					Equal IM and SS	
					Control vs resveratrol	0.129173

Extended Data Table 10: Statistical tests for experiments in presented Extended Data Figures

Figure	Description	Test used	t/F value	degrees of freedom (df)	Multiple comparison	p values (adjusted)
Extended 3a	PC levels	One way ANOVA with Tukey's multiple correction post hoc test	8.782	17	22 week vs. 37 week	0.0266
					22 week vs. 52 week	0.4007
					37 week vs. 52 week	0.0018
Extended 3b	PE levels	One way ANOVA with Tukey's multiple correction post hoc test	10.93	17	22 week vs. 37 week	0.0121
					22 week vs. 52 week	0.0009
					37 week vs. 52 week	0.751
Extended 3c	PI levels	One way ANOVA with Tukey's multiple correction post hoc test	7.909	17	22 week vs. 37 week	0.0172
					22 week vs. 52 week	0.7406
					37 week vs. 52 week	0.0035
Extended 3d	PS levels	One way ANOVA with Tukey's multiple correction post hoc test	9.227	17	22 week vs. 37 week	0.0016
					22 week vs. 52 week	0.0375
					37 week vs. 52 week	0.1777
Extended 3e	PG levels	One way ANOVA with Tukey's multiple correction post hoc test	6.375	16	22 week vs. 37 week	0.7169
					22 week vs. 52 week	0.0097
					37 week vs. 52 week	0.0726
Extended 3f	Ceramide levels	One way ANOVA with Tukey's multiple correction post hoc test	18.01	17	22 week vs. 37 week	0.0004
					22 week vs. 52 week	0.7121
					37 week vs. 52 week	<0.0001
Extended 3g	Acyl carnatines	Chi squared test	8.129 (Chi-square)	2	N/A	0.0172
Extended 3h	Glutathione	One way ANOVA with Tukey's multiple correction post hoc test	0.1967	17	22 week vs. 37 week	0.8832
					22 week vs. 52 week	0.8303
					37 week vs. 52 week	0.9991
Extended 4f	Myosin 7	Two way ANOVA with Tukey's multiple correction post hoc test	-	23	22 week vs. 37 week	0.3276
					22 week vs. 52 week	0.6687
					37 week vs. 52 week	0.8648
Extended 4f	Myl2/3	Two way ANOVA with Tukey's multiple correction post hoc test	-	23	22 week vs. 37 week	0.6356
					22 week vs. 52 week	0.5187
					37 week vs. 52 week	0.9799
Extended 5a	Diglyceride levels	Unpaired t test (one-tailed)	0.3184	11	Control vs resveratrol	0.3781
Extended 5b	CDP-choline	Unpaired t test (one-tailed)	1.367	12	Control vs resveratrol	0.0984
Extended 5c	PC levels	Unpaired t test (one-tailed)	1.802	11	Control vs resveratrol	0.0495

Extended 5d	PE levels	Unpaired t test (one-tailed)	1.941	11	Control vs resveratrol	0.0392
Extended 5e	PI levels	Unpaired t test (one-tailed)	0.9185	12	Control vs resveratrol	0.1882
Extended 5f	PS levels	Unpaired t test (one-tailed)	4.121	12	Control vs resveratrol	0.0007
Extended 5g	PG levels	Unpaired t test (one-tailed)	2.789	11	Control vs resveratrol	0.0088
Extended 5h	Ceramide levels	Unpaired t test (one-tailed)	0.852	12	Control vs resveratrol	0.2055
